# Antiobesity and antidiabetic effects of the dairy bacterium *Propionibacterium freudenreichii* MJ2 in high-fat diet-induced obese mice by modulating lipid metabolism

**DOI:** 10.1038/s41598-021-82282-5

**Published:** 2021-01-28

**Authors:** Mirae An, Yeon-Hee Park, Young-Hee Lim

**Affiliations:** 1 Department of Healthcare Sciences, Graduate School, Korea University, Seoul, 02841 Republic of Korea; 2 BK21FOUR R&E Center for Learning Health Systems, Korea University, Seoul, 02841 Republic of Korea; 3 Department of Public Health Science, Graduate School, Korea University, Seoul, 02841 Republic of Korea; 4 Department of Integrated Biomedical and Life Sciences, Graduate School, Korea University, Seoul, 02841 Republic of Korea; 5 Department of Laboratory Medicine, Korea University Guro Hospital, Seoul, 08308 Republic of Korea

**Keywords:** Microbiology, Diseases

## Abstract

Obesity can cause chronic metabolic disorders such as type 2 diabetes, hyperlipidemia, and nonalcoholic fatty liver diseases. The aim of this study was to investigate the antiobesity and antidiabetic effects of the dairy bacterium *P. freudenreichii* MJ2 isolated from raw milk using 3T3-L1 cells and high-fat diet (HFD)-induced obese mice. Lipid accumulation and the expression levels of genes related to lipid metabolism, such as preadipocytic gene (*Pref-1*), adipogenic genes (*PPARγ* and *C/EBPα*), and lipogenic genes (*FAS*, *SCD-1*, and *ACC*), significantly decreased in heat-killed *P. freudenreichii* MJ2 (hkMJ2)-treated adipocytes. Live *P. freudenreichii* MJ2 (MJ2), hkMJ2, and *Lactobacillus plantarum* (LP) decreased body weight gain in HFD-induced obese mice compared with the model group. The liver and epididymal white adipose tissue weights in the MJ2-, hkMJ2- and LP-treated groups were significantly lower than those in the model group. The expression levels of genes and proteins related to adipogenesis and lipogenesis significantly decreased and lipolysis (HSL and ATGL) increased in the MJ2-, hkMJ2-, and LP-treated groups. The expression levels of genes related to fatty acid β-oxidation (*CPT-1α* and *ACOX1*) increased in the MJ2-, hkMJ2-, and LP-treated groups. In addition, blood glucose and fasting insulin levels in the MJ2- and hkMJ2-treated groups decreased compared with those in the model group. *P. freudenreichii* MJ2 ameliorate insulin resistance by obesity. In conclusion, both MJ2 and hkMJ2 alleviate obesity and metabolic syndrome.

## Introduction

Obesity, defined as a body mass index over 30, is considered a major factor that induces not only metabolic syndromes such as type 2 diabetes by increasing insulin resistance, hyperlipidemia, and liver diseases including nonalcoholic fatty liver disease (NAFLD) but also diverse diseases including obstructive sleep apnea, depression, and some kinds of cancer^1^. In particular, insulin resistance is the crucial pathogenic determinant that induces other metabolic disorders^2^. Obesity is a metabolic complex disease; thus, there are various causes of obesity and antiobesity mechanisms, such as decreased lipid absorption, appetite suppressants, inhibition of preadipocyte differentiation, and increased energy expenditure. Many drugs are being developed due to the serious risks of obesity, but most drugs for improving obesity are not used long term because of lack of effectiveness or serious side effects such as vomiting, constipation, insomnia, and diarrhea^3^. Orlistat, a representative drug known to be safe, relieves obesity by inhibiting pancreatic and gastric lipase, which decomposes triglycerides to the prevent absorption of free fatty acids. Although the safety of orlistat has been proven, limitations remain, such as steatorrhea with excessive flatus^4^, and the mechanism of alleviating overweight is only partially understood.

Recently, awareness of the importance of the composition of gut microbiota has increased with the revelation that various diseases are associated with dysbiosis, that is, a microbial imbalance inside the body. Although the alteration of the gut microbiota by obesity is not clearly explained, dysbiosis and obesity might be correlated^5^. When dysbiosis occurs with obesity, major species of gastrointestinal microbiota and their beneficial metabolites, such as short chain fatty acids (SCFAs), vitamin B_12_, and indole, are lost, and intestinal permeability and endotoxemia are increased^6^, which induces inflammation and gluconeogenesis in the liver, decreases satiety in the brain, and increases triglyceride incorporation and inflammation in adipose tissues. In addition, increased gut permeability maintains low-grade inflammation, and such chronic inflammation induces obesity^7^. Individual or multiple strains of probiotics have been actively studied to improve obesity. The definition of probiotics, a live organism that can prevent, treat, and cure diseases in the host when administered at adequate concentrations, was established by the FAO/WHO (Food and Agriculture Organization/World Health Organization) in 2002. In addition to the diverse treatment effects of probiotics on allergies, inflammatory bowel disease, immune function, and obesity, probiotics can alter fatty acid metabolism and stimulate glycolysis in obese individuals^8^. However, the established probiotic bacterial strains include only a narrow range of organisms; thus, according to developing knowledge of the gut microbiota, the development of novel microbial therapeutic organisms that show beneficial effects on humans is necessary^9^.

*Propionibacterium freudenreichii* is a gram-positive bacterium usually used to produce fermented dairy products, especially in ripening culture in the manufacture of Swiss-type cheese. As a dietary fermenter, it is also in the Generally Recognized As Safe (GRAS) with *Lactobacilli* and *Bifidobacteria,* which represent the majority of the probiotics being applied industrially. In addition, *P. freudenreichii* is considered a beneficial bacterium that produces bifidogenic compounds that promote the growth of *Bifidobacterium* spp. It produces vitamin B_12_, propionic acid, and surface proteins that show immunomodulatory properties^10,11^. According to these features, *P. freudenreichii* is considered a next-generation probiotic that can confer health benefits on the host. Therefore, in this study, we investigated the probiotic effect of *P. freudenreichii* MJ2 isolated from raw milk on the prevention of lipid accumulation in adipocytes and its antiobesity and antidiabetic activity in high-fat diet (HFD)-induced obese mice.

## Results

### Effect of heat-killed *P. freudenreichii* MJ2 (hkMJ2) on the cell viability of 3T3-L1 preadipocytes

The 3T3-L1 preadipocyte cell line has the potential to differentiate from fibroblasts to an adipocyte-like phenotype^12^. To investigate the cytotoxic effect of hkMJ2 on 3T3-L1 cells, various concentrations of hkMJ2 (10^5^, 10^6^, 10^7^, and 10^8^ cells/mL) were applied to 3T3-L1 cells for 24 h, and cell viability was determined by MTT assay. The results showed no significant differences in the cell viability of 3T3-L1 preadipocytes between all hkMJ2-treated cells and the negative control (Supplementary Fig. 1). Therefore, hkMJ2 did not show any cytotoxicity within the concentrations used in this study. Based on these results, the cellular experiments in this study were performed at concentrations of 10^6^ cells/mL, 10^7^ cells/mL, and 10^8^ cells/mL hkMJ2.

**Figure 1:**
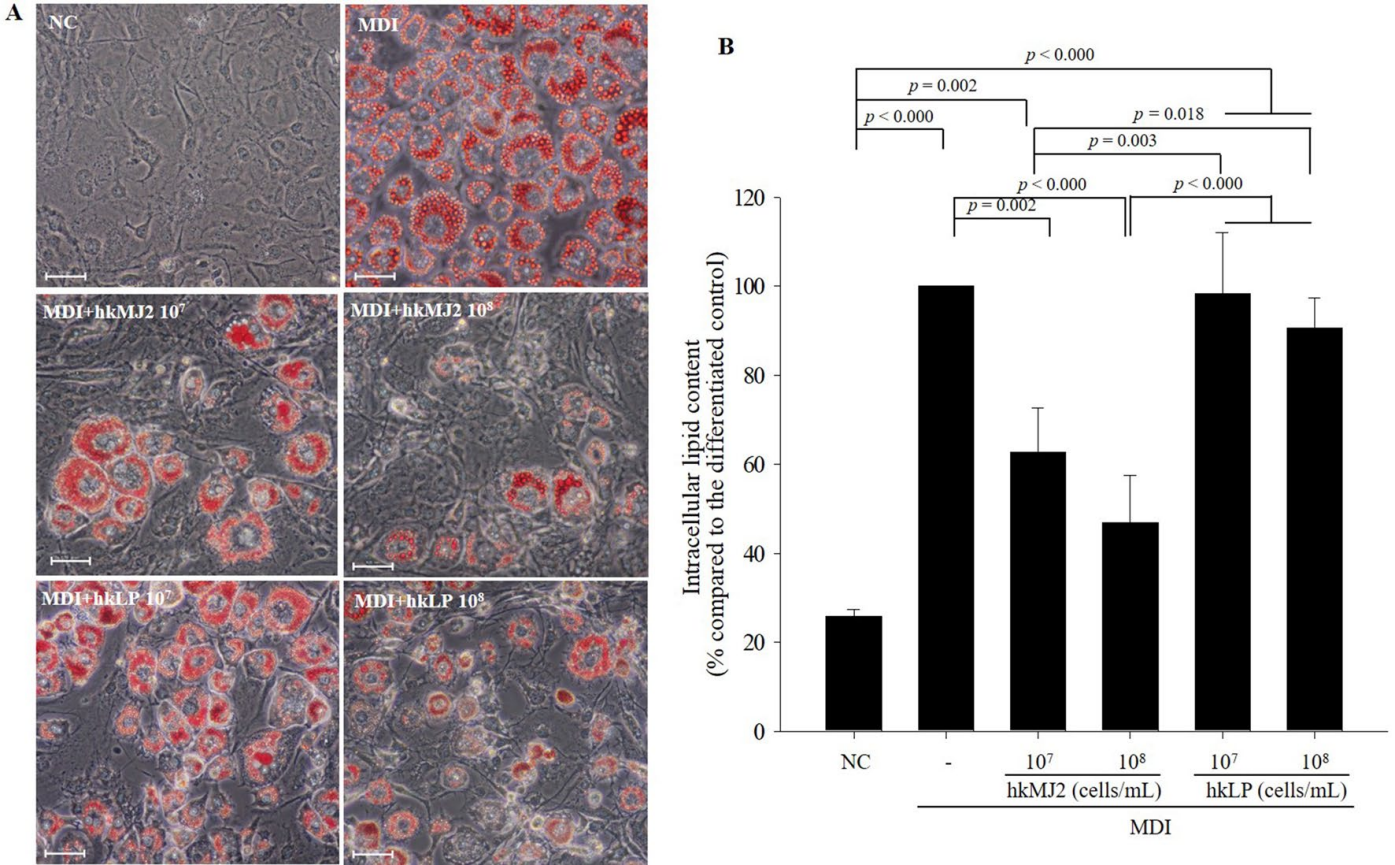
Inhibitory effects of hkMJ2 and hkLP on lipid accumulation in 3T3-L1 adipocytes. 3T3-L1 preadipocytes were differentiated by treatment with differentiation-inducing medium containing MDI (IBMX + dexamethasone + insulin), and heat-killed *P. freudenreichii* MJ2 (hkMJ2) or heat-killed *L. plantarum* (hkLP) were simultaneously administered at the designated concentrations during the period of differentiation. (**A**) Lipid droplets of the differentiated cells were visualized by oil red O staining (200× magnification) and the scale bars indicate 0.25 mm. (**B**) Lipid accumulation was quantified by measuring oil droplets stained with oil red O and comparing them with cells treated with MDI alone. The data indicate the mean ± SD of three independent experiments. The* p* values are determined by ANOVA and Tukey’s HSD test.

### hkMJ2 suppressed lipid accumulation during the differentiation of preadipocytes into adipocytes

The differentiation of 3T3-L1 preadipocytes was induced by treatment with differentiation-inducing medium containing MDI (3-isobutyl-1-methylxanthine (IBMX) + dexamethasone + insulin). To examine the effect of hkMJ2 on the inhibition of lipid accumulation in 3T3-L1 cells, hkMJ2 (10^6^, 10^7^, and 10^8^ cells/mL) was administered during the induction of differentiation. Lipid accumulation in the hkMJ2-treated cells decreased in a dose dependent manner compared with the cells treated with MDI alone (100%) (Supplementary Fig. 2). In addition, the effect of hkMJ2 on the inhibition of lipid accumulation in 3T3-L1 cells was compared with heat-killed *Lactobacillus plantarum* (hkLP). *L. plantarum* is a representative probiotic that has an antiobesity effect in a HFD-induced obese mouse model and inhibits lipid accumulation in maturing preadipocytes by modulating adipogenesis^13–15^. Thus, *L. plantarum* was used as a comparable control in this study. Lipid accumulation was quantified by staining the produced triglyceride lipid droplets with oil red O. The relative lipid accumulations (%) were 25.8 ± 1.62%, 62.7 ± 10.0%, 46.9 ± 10.5%, 98.4 ± 13.7%, and 90.6 ± 6.7% in the negative control 3T3-L1 cells and those treated with 10^7^ cells/mL hkMJ2, 10^8^ cells/mL hkMJ2, 10^7^ cells/mL hkLP, and 10^8^ cells/mL hkLP, respectively, compared with 3T3-L1 cells treated with MDI alone (100%) (Fig. 1). Lipid accumulation significantly increased when the differentiation of preadipocytes into adipocytes was induced. However, lipid droplets were significantly reduced in adipocytes treated with hkMJ2 in a dose-dependent manner compared with adipocytes without treatment. In particular, hkMJ2 significantly inhibited the production of lipid droplets compared to that in adipocytes treated with hkLP. Therefore, the results suggested that hkMJ2 possesses antiobesity potential.

**Figure 2:**
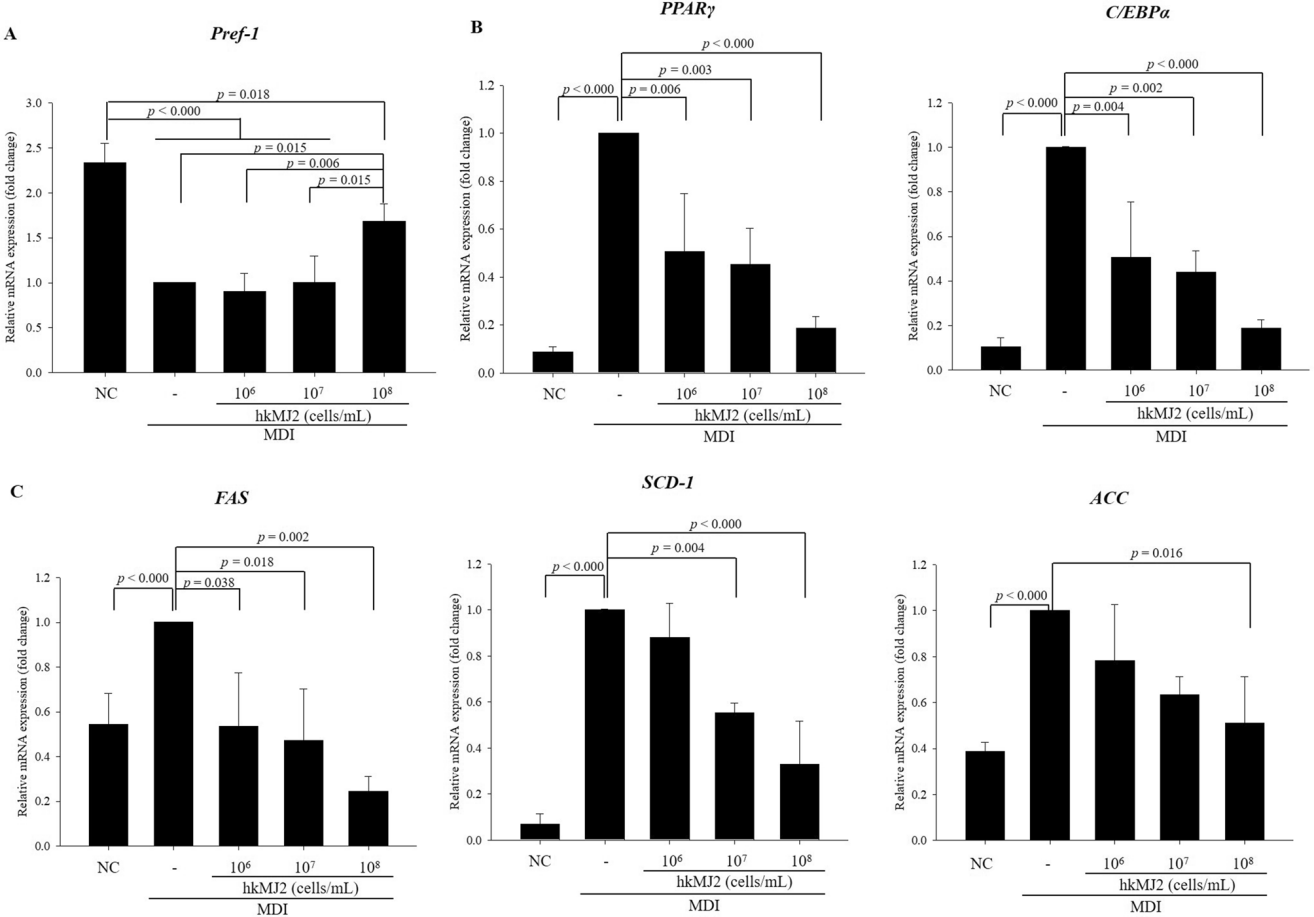
Effect of hkMJ2 on the expression levels of genes related to adipogenesis and lipogenesis. mRNA expression levels in 3T3-L1 adipocytes were analyzed by qPCR. 3T3-L1 preadipocytes were differentiated with differentiation-inducing medium containing MDI (IBMX + dexamethasone + insulin), and each concentration of hkMJ2 was tested. The treatment was maintained during the period of differentiation. (**A**) Preadipocyte factor-1 (*Pref-1*) in 3T3-L1 adipocytes. (**B**) Adipogenic transcription factors (*PPARγ* and *C/EBPα*) in 3T3-L1 adipocytes. (**C**) Lipogenic transcription factors *(FAS*, *SCD-1* and *ACC*) in 3T3-L1 adipocytes. The data indicate the mean ± SD of three independent experiments. The* p* values were determined by ANOVA and Tukey’s HSD test.

### hkMJ2 reduced the expression levels of genes related to adipogenesis and lipogenesis in differentiated adipocytes

During differentiation, preadipocytes acquire the adipocyte phenotype, including changes in morphology and cytoskeletal components and chronological changes in gene expression^16^. Preadipocyte factor-1 (Pref-1), a transmembrane protein identified in 3T3-L1 preadipocyte, plays an important role in maintaining the preadipocyte state. Thus, the expression level of Pref-1 decreases during adipocyte differentiation. The expression of peroxisome proliferator-activated receptor gamma (PPARγ) and CCAAT/enhancer-binding protein alpha (C/EBPα) increases during the early differentiation period and plays critical roles during the adipogenic period^17^. In late differentiation period, triacylglycerols are produced by differentiated adipocytes by the increase of the expression of lipogenic factors such as fatty acid synthase (FAS), stearoyl-CoA desaturase-1 (SCD-1), and acetyl-CoA carboxylase (ACC)^18^. To investigate the effect of hkMJ2 on the inhibition of differentiation and lipogenesis, mRNA expression levels related to adipogenesis and lipogenesis were measured by qPCR. The expression levels of *Pref-1* in the MDI-treated groups significantly decreased compared with the NC and the expression level of *Pref-1* in adipocytes treated with hkMJ2 (10^8^ cells/mL) significantly increased compared with that in untreated adipocytes (the cells treated with MDI alone) (Fig. 2A). The expression levels of *PPARγ* and *C/EBPα* in adipocytes treated with hkMJ2 (10^6^, 10^7^, and 10^8^ cells/mL) significantly decreased in a dose dependent manner compared with those in untreated adipocytes (Fig. 2B). The expression levels of adipogenic genes (*FAS*, *SCD-1*, and *ACC*) in adipocytes decreased by treatment with hkMJ2 (10^6^, 10^7^, and 10^8^ cells/mL) in a dose-dependent manner compared with those in untreated adipocytes (Fig. 2C). The results showed that the reduction in lipid accumulation in adipocytes treated with hkMJ2 might be due to a decrease in the expression levels of lipogenic genes by treatment with hkMJ2.

### Effect of live *P. freudenreichii* MJ2 (MJ2) and hkMJ2 on body, liver, and epididymal white adipose tissue (eWAT) weight changes in HFD-induced obese mice

**Figure 3:**
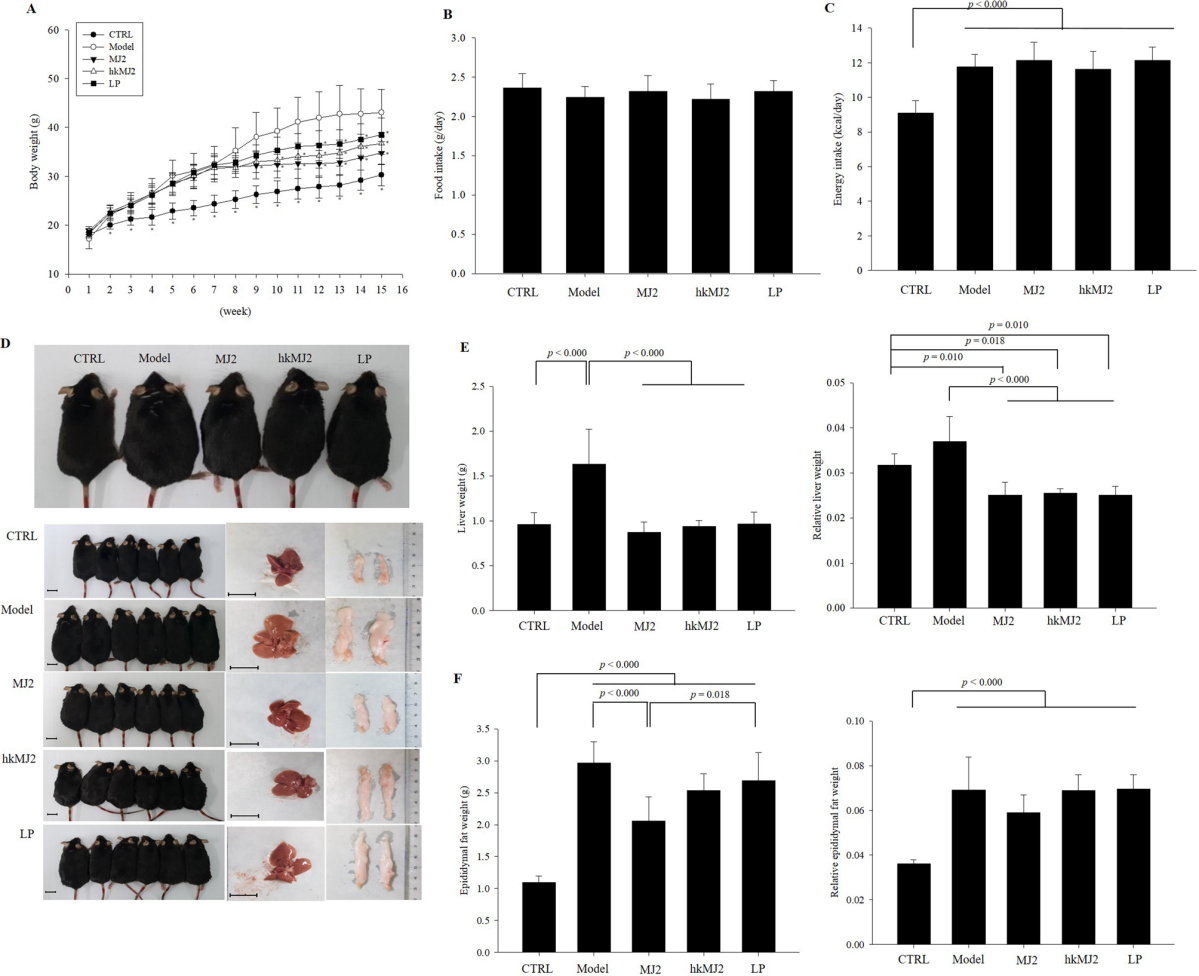
Effects of MJ2 and hkMJ2 on body weight, liver weight and eWAT weight changes in HFD-induced obese mice (n = 6/group). C57BL/6N mice were induced to develop obesity by feeding HFD for 7 weeks followed by feeding with HFD and MJ2, hkMJ2 or LP for 8 weeks. (**A**) Body weight was measured weekly. The data indicate the mean ± SD (*p < 0.05 compared with the model). (**B**) Food intake and (**C**) energy intake were measured weekly. (**D**) Representative photographs of mice, eWAT and liver of each group. The scale bars indicate 1.5 cm. The liver (**E**) and epididymal fat (**F**) were washed and weighed immediately after sacrifice. The data indicate the mean ± SD, and the* p* values were determined by ANOVA and Tukey’s HSD test.

To assess the antiobesity effect of *P. freudenreichii *in vivo, live *P. freudenreichii* MJ2 (MJ2), hkMJ2, or live *L. plantarum* (LP) was orally administered to HFD-induced obese mice. LP was used as a control group. The body weights of the HFD-fed groups significantly increased compared with those of the normal diet-fed control (CTRL) (Fig. 3A), while food intake (g/day) did not show a significant difference between the groups during the experiment (Fig. 3B). After 8 weeks of treatment with MJ2, hkMJ2, or LP, body weights in the MJ2, hkMJ2, and LP groups significantly decreased by 31%, 22.8%, and 18.5%, respectively, compared with the model group. Energy intake (kcal/day) of the HFD-fed groups was significantly higher than that of the CTRL and it did not significantly different between the HFD-fed groups (Fig. 3C). The food efficiency ratio (FER) of the MJ2, hkMJ2, and LP groups significantly decreased the ratio compared with the model group (Supplementary Table 1). Although the oral administration of MJ2, hkMJ2, and LP did not affect the amount of food intake and energy intake, body weights decreased in the MJ2, hkMJ2, and LP groups, which indicates that the decrease in body weight was not derived from a reduction in food intake and energy intake in those groups. This also affected the decrease in FERs in those groups.

The liver weights and the relative liver weights (liver weight/body weight) in the MJ2, hkMJ2, and LP groups significantly decreased compared with those in the model group (Fig. 3D,E). Unlike the liver weight, eWAT weight decreased significantly only in the MJ2 group compared with the model group, and relative eWAT weight (eWAT weight/body weight) did not show any significant difference in any groups except the CTRL (Fig. 3D,F). The results showed that MJ2, hkMJ2, and LP specifically inhibit fat accumulation in the liver, and both live and heat-killed MJ2 show a similar prevention effect with the comparable control, LP.

### MJ2 and hkMJ2 suppressed the expression of genes and proteins related to adipogenesis, lipogenesis, lipolysis, and fatty acid β-oxidation in eWAT

**Figure 4:**
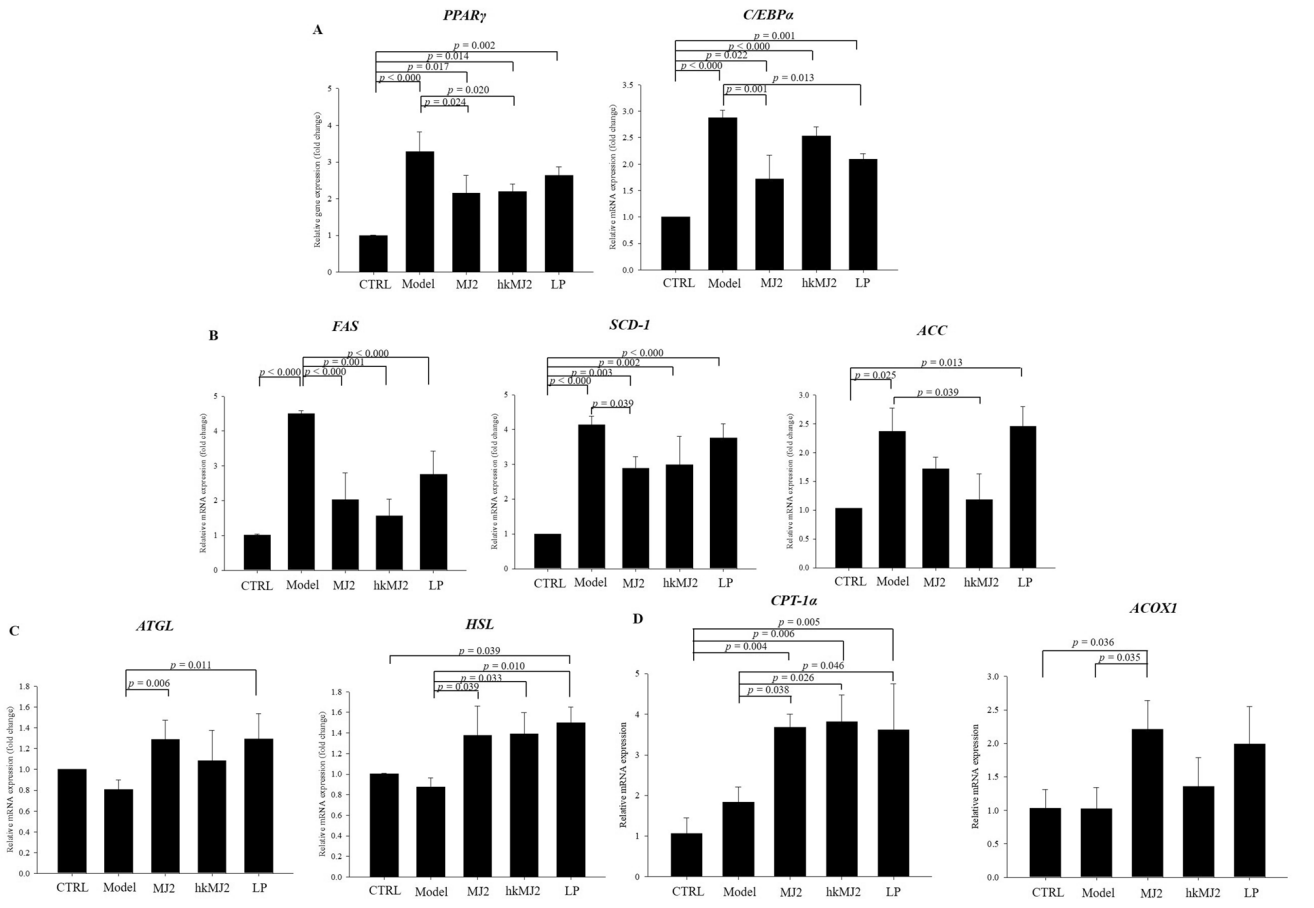
Effects of MJ2 and hkMJ2 on adipogenesis, lipogenesis, lipolysis, and fatty acid β-oxidation in eWAT. The mRNA was extracted from the eWAT of each group, and gene expression levels of (**A**) adipogenic (*PPARγ* and *C/EBPα*), (**B**) lipogenic (*FAS*, *SCD-1*, and *ACC*), (**C**) lipolytic (*ATGL* and *HSL*), and (**D**) fatty acid β-oxidation (*CPT-1* and *ACOX1*) factors related to lipid metabolism were analyzed by qPCR. The data indicate the mean ± SD. The* p* values were determined by ANOVA and Tukey’s HSD test.

eWAT was used to investigate the inhibitory effects of MJ2 and hkMJ2 on adipogenesis, lipogenesis, and lipolysis in adipocytes. The mRNA expression levels of adipogenic factors significantly increased in the model group compared with the CTRL. The expression levels of *PPARγ* in the MJ2 and hkMJ2 significantly decreased; the expression levels of *C/EPBα* in the MJ2 and LP groups significantly decreased compared with those in the model group (Fig. 4A). The mRNA expression levels of lipogenic factors significantly increased in the model group compared with the CTRL. The expression levels of *FAS*, *SCD-1*, and *ACC* in the MJ2, hkMJ2, and LP groups decreased compared with those in the model group (Fig. 4B). The rate of lipid accumulation is determined by the balance between the lipogenesis and lipolysis pathways^19^. Lipolytic enzymes, such as adipose tissue triglyceride lipase (ATGL) and hormone-sensitive lipase (HSL), hydrolyze intracellular triglycerides to glycerol and free fatty acids^20^. Interestingly, the expression levels of lipolytic factors did not show a significant difference between the model and CTRL (Fig. 4C). However, the expression levels of *ATGL* and *HSL* in the MJ2, hkMJ2, and LP groups increased compared with those in the model group. In addition, the effect of MJ2 on carnitine palmitoyltransferase 1α (CPT-1α) and peroxisomal acyl-coenzyme A oxidase 1 (ACOX1) related to fatty acid β-oxidation was investigated. Expression levels of *CPT-1α* and *ACOX1* increased in the MJ2, hkMJ2, and LP groups compared with those in the model group (Fig. 4D). The overall results showed that oral administration of MJ2, hkMJ2, and LP inhibited adipogenesis and lipogenesis, which was consistent with the in vitro results, and accelerated lipolysis and fatty acid β-oxidation in eWAT.

**Figure 5:**
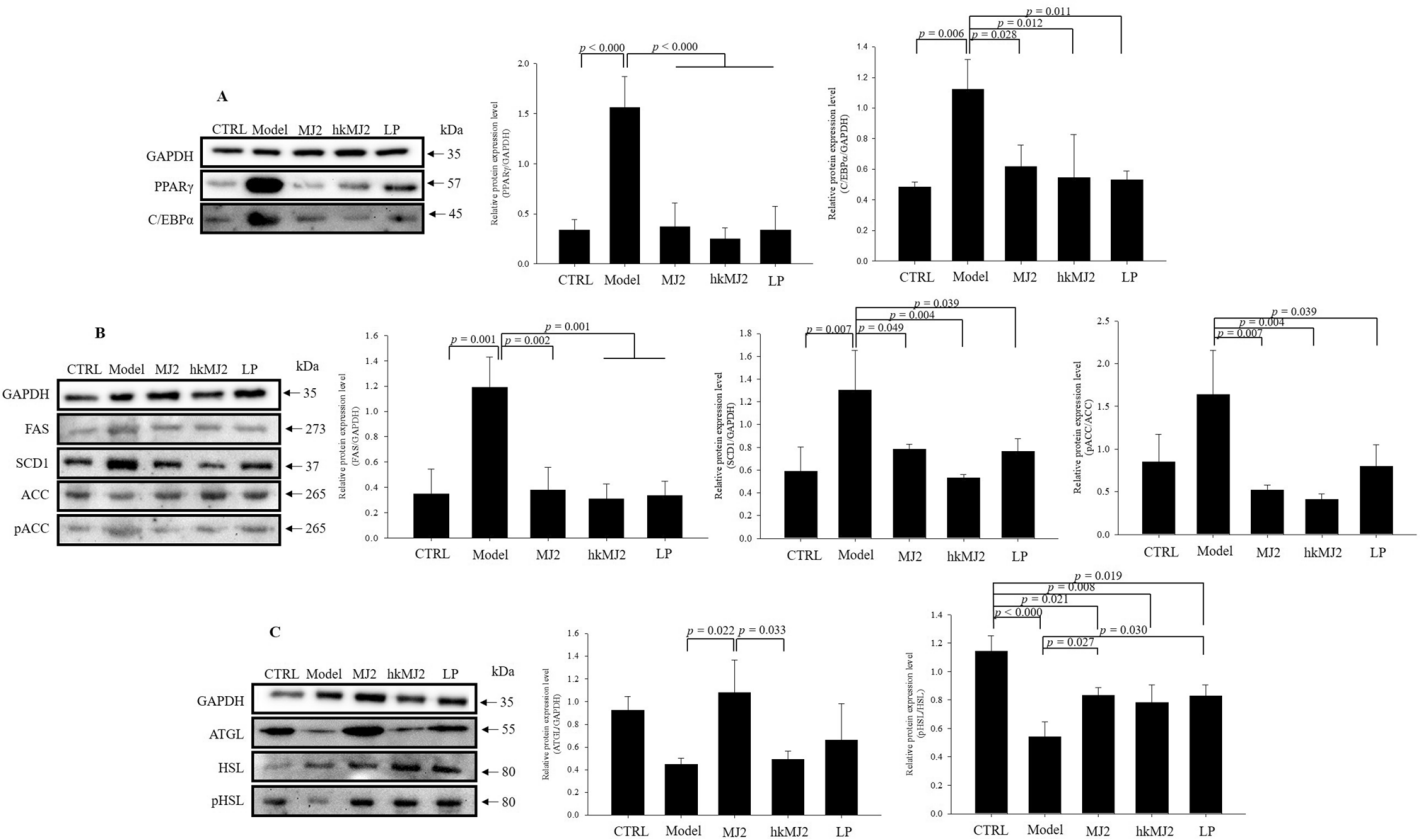
Effects of MJ2 and hkMJ2 on protein expression levels related to adipogenesis and lipid metabolism in eWAT. Total protein was extracted from eWAT in each group, and the relative protein expression levels of the factors related to (**A**) adipogenesis (PPARγ and C/EBPα), (**B**) lipogenesis (FAS, SCD-1 and ACC) and (**C**) lipolysis (ATGL and HSL) were investigated by western blot. Representative images of each protein are shown, and the relative quantified expression levels indicate the mean ± SD. The* p* values were determined by ANOVA and Tukey’s HSD test. The full-length blots are shown in Supplementary Fig. 3.

To investigate the protein expression levels related to adipogenesis and lipid metabolism, total protein was extracted from eWAT in each group, and western blot analysis was performed. The protein expression levels of adipogenic factors significantly increased in the model group compared with the CTRL (Fig. 5A). The relative expression levels of PPARγ in the MJ2, hkMJ2, and LP groups significantly decreased 0.24-, 0.16-, and 0.22-fold, respectively; and the relative expression levels of C/EBPα in the MJ, hkMJ2, and LP groups significantly decreased 0.55-, 0.48-, and 0.47-fold, respectively, compared with those in the model group. The protein expression levels of lipogenic factors significantly increased in the model group compared with the CTRL (Fig. 5B). The relative expression levels of FAS in the MJ2, hkMJ2, and LP groups significantly decreased 0.32-, 0.26-, and 0.28-fold, respectively; the relative expression levels of SCD-1 in the MJ2, hkMJ2, and LP groups significantly decreased 0.60-, 0.41-, and 0.59-fold, respectively; and the relative expression levels of phospho-ACC (pACC)/ACC in the MJ2, hkMJ2, and LP groups significantly decreased 0.36-, 0.27-, and 0.51-fold, respectively, compared with those in the model group. The protein expression levels of lipolytic factors decreased in the model group compared with the CTRL, which was not consistent with the mRNA expression results (Fig. 5C). In particular, the relative expression level of ATGL in the MJ2 group significantly increased 2.42-fold compared with the model group, while the hkMJ2 and LP groups did not show a significant increase. Unlike ATGL, HSL is activated by phosphorylation followed by translocation to the lipid droplet where triglycerides are stored^21^. The relative expression levels of pHSL/HSL in the MJ2, hkMJ2, and LP groups increased 1.55-, 1.45-, and 1.54-fold, respectively, compared with those in the model group. These results showed that the general expression tendency of each factor was consistent with the gene expression. The results suggested that MJ2 and hkMJ2 effectively regulated the expression of genes and proteins related to adipogenesis and lipid metabolism to inhibit lipid production.

### MJ2 and hkMJ2 reduced adipocyte size in eWAT

**Figure 6:**
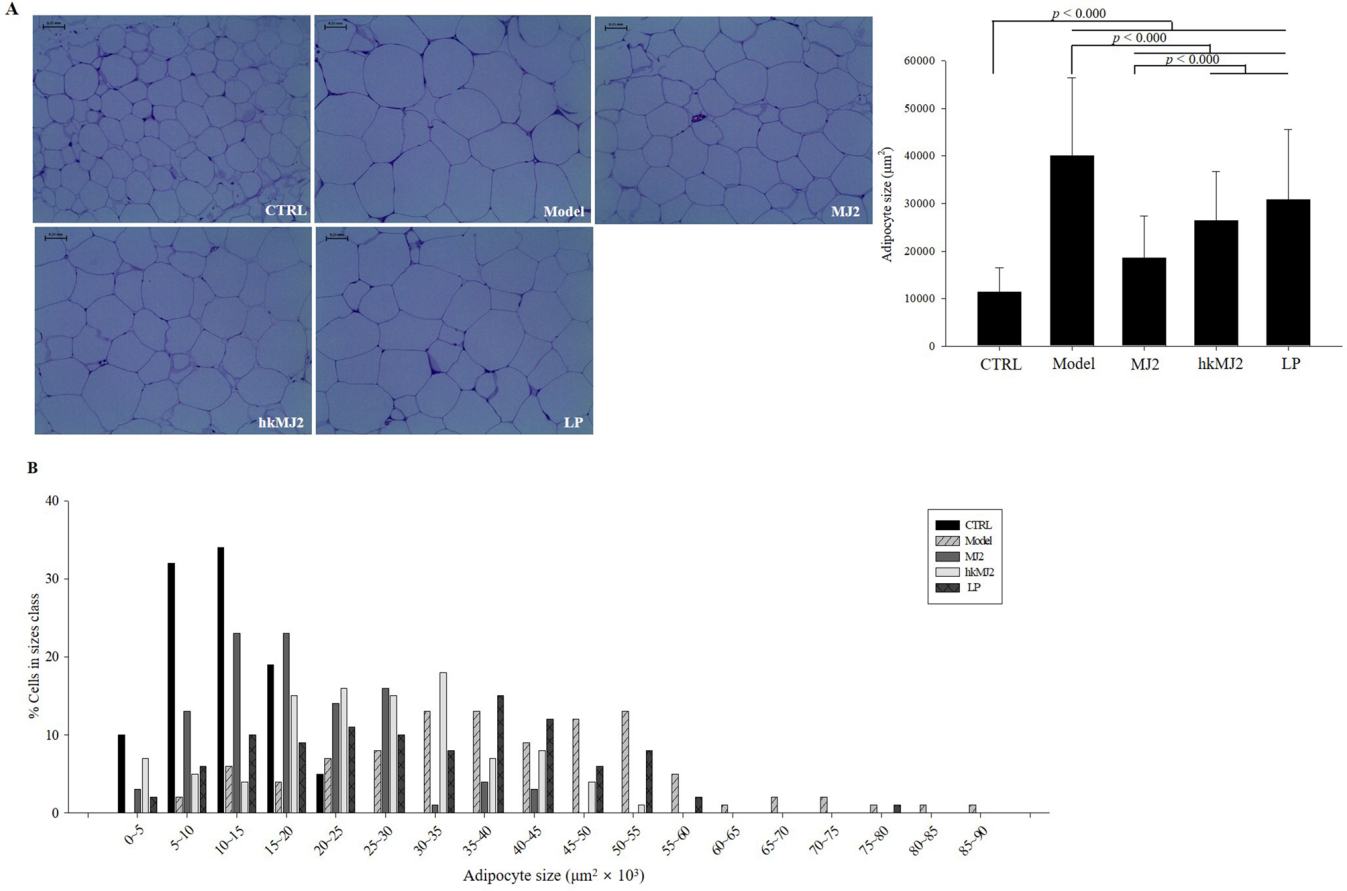
Effects of MJ2 and hkMJ2 on eWAT adipocyte size. The size of adipocytes in eWAT was investigated by H&E staining. (**A**) Representative H&E staining of adipocytes in eWAT and (**B**) size distribution of adipocytes (n = randomly selected 100 cells/group, at least 4 parts were evaluated for each sample). The scale bars indicate 0.25 mm, and the slides were observed at 200× magnification. The data indicate the mean ± SD, and the* p* values were determined by ANOVA and Tukey’s HSD test.

The effects of MJ2 and hkMJ2 on adipocyte size in HFD-induced obese mice was investigated by hematoxylin and eosin (H&E) staining of eWAT. The size of adipocytes significantly increased in the model group compared with the CTRL (Fig. 6A). However, oral treatment with MJ2, hkMJ2, or LP significantly decreased adipocyte size. The adipocyte sizes in the MJ2, hkMJ2, and LP groups significantly decreased by 53.5%, 33.9%, and 23.2%, respectively, compared with the model group. In particular, the MJ2 group had significantly decreased adipocyte size compared with those in the hkMJ2 and LP groups. The distribution according to cell size also showed that the cell numbers of higher size were reduced in the MJ2, hkMJ2, and LP groups (Fig. 6B). The cell sizes of the MJ2 and hkMJ2 groups were below 55 × 10^3^ μm^2^. The results showed that MJ2 and hkMJ2 inhibited the extension of adipocytes in HFD-fed mice.

### MJ2 positively affected serum lipids in HFD-induced obese mice

To determine the effects of MJ2, hkMJ2, and LP on serum lipids, the serum levels of total cholesterol (TCHO), high-density lipoprotein cholesterol (HDL), low-density lipoprotein cholesterol (LDL), and triglycerides (TG) were measured in the serum collected on the last day of the experiment. The TCHO level significantly increased in the model group compared with the CTRL, while it significantly decreased in the MJ2 group compared with the model group (Supplementary Table 2). TG levels did not show significant differences between the groups. LDL level also significantly increased in the model group compared with the CTRL, while it significantly decreased in the MJ2 group compared with the model group. Unexpectedly, the HDL level in the model group significantly increased compared with that in the CTRL, while it significantly decreased in the MJ2 group compared with that in the model group, and the level was similar to that in the CTRL. Although the hkMJ2- and LP-treated groups tended to show slightly decreased serum levels of TCHO, HDL, and LDL compared with the model group, there was no significant difference between the model group and the hkMJ2 and LP groups. Although the serum level of HDL in this study increased in the model group, the ratio of HDL serum level to the TCHO in serum was 37.3%, 37.5%, 75.7%, 73.1%, and 74.1% in the CTRL, model, MJ2, hkMJ2, and LP groups, respectively. Regardless of whether the ratio of serum HDL level to TCHO in the model group was similar to that in the CTRL, the ratio of serum HDL level to TCHO markedly increased in the MJ2, hkMJ2, and LP groups. The results showed that the administration of MJ2, hkMJ2, and LP reduced blood TCHO and LDL and increased the level of HDL in HFD-fed mice.

### MJ2 and hkMJ2 alleviated insulin resistance in HFD-induced obese mice

**Figure 7:**
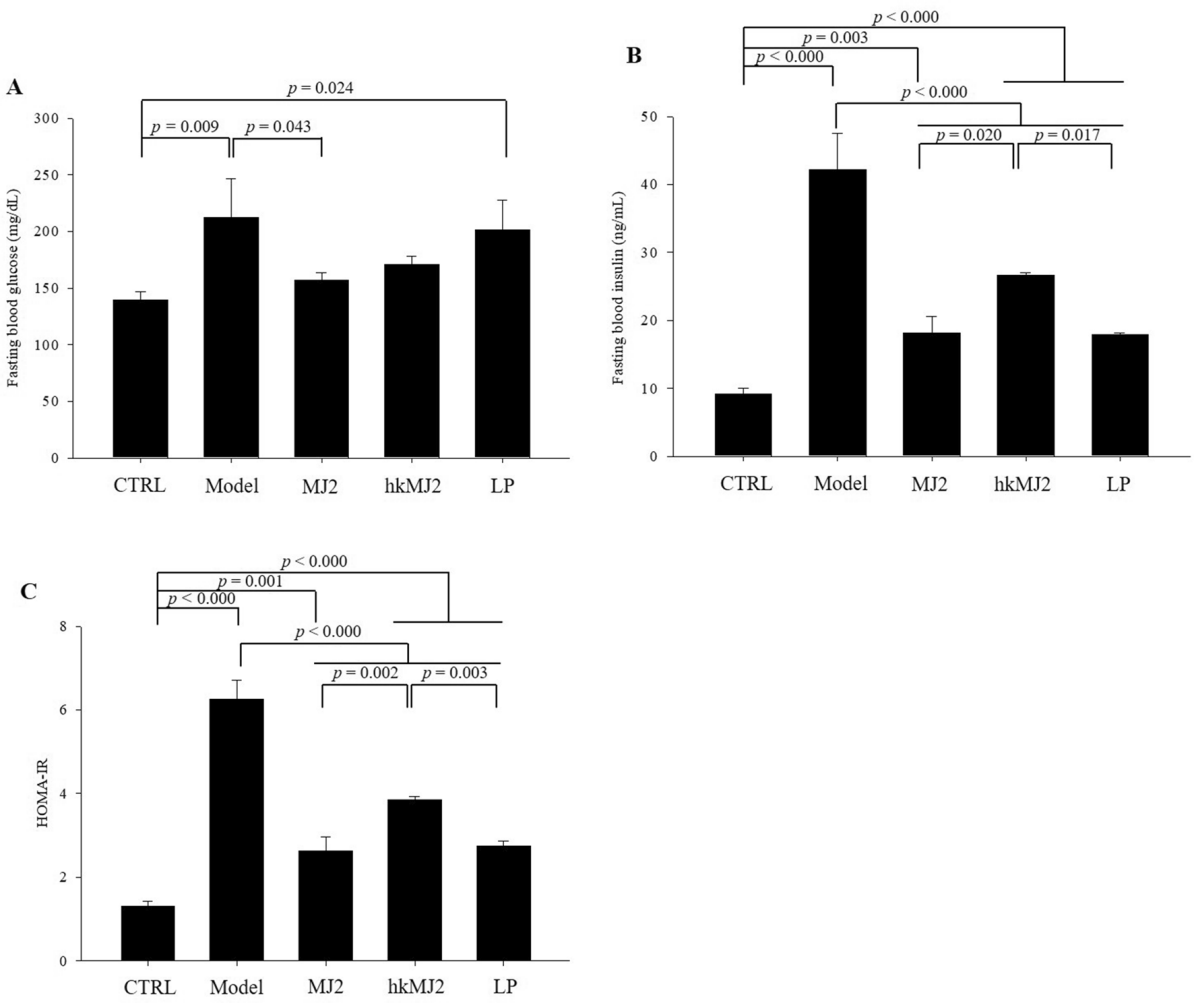
Effects of MJ2 and hkMJ2 on blood glucose (**A**), fasting blood insulin (**B**) and HOMA-IR (**C**) in HFD-induced obese mice (n = 6/group). The blood glucose and fasting insulin levels were measured by using Accu-Chek and ELISA, respectively. The HOMA-IR score was calculated by the HOMA2 calculator. The data indicate the mean ± SD, and the* p* values were determined by ANOVA and Tukey’s HSD test.

Obesity is a major cause of diverse metabolic disorders. The increase in obesity may be the most perilous factor in type 2 diabetes because of increased insulin resistance related to the secretion of nonesterified fatty acids in obese adipose tissue and the dysfunction of pancreatic β-cells^22^. To investigate the alleviating effects of MJ2 and hkMJ2 on insulin resistance in HFD-induced obese mice, blood glucose and insulin were measured, and HOMA-IR was calculated to determine the presence and extent of insulin resistance. The blood glucose level of the model group (212 ± 19.9 mg/dL) was significantly increased compared with that of the CTRL (139.7 ± 7 mg/dL) (Fig. 7A). The blood glucose level was significantly reduced in the MJ2 group compared with the model group. The blood glucose levels were 156.7 ± 6.7 mg/dL, 171 ± 7.2 mg/dL, and 201.3 ± 25.9 mg/dL in the MJ2, hkMJ2, and LP groups, respectively. The fasting blood insulin level in the model group (1.46 ± 0.19 ng/mL) was significantly increased compared with that in the CTRL (0.32 ± 0.03 ng/mL) (Fig. 7B). On the other hand, the fasting blood insulin levels in the MJ2, hkMJ2, and LP groups significantly decreased compared with those in the model group. The fasting blood insulin levels were 0.63 ± 0.08 ng/mL, 0.92 ± 0.01 ng/mL, and 0.62 ± 0.01 ng/mL in the MJ2, hkMJ2, and LP groups, respectively. The HOMA-IR score of the model group significantly increased 4.8-fold compared with that of the CTRL (Fig. 7C). The HOMA-IR scores of the CTRL and model groups were 1.3 ± 0.1 and 6.3 ± 0.5, respectively, which showed that the CTRL had optimal insulin sensitivity; however, the model group exhibited significant insulin resistance because of HFD feeding. On the other hand, the HOMA-IR scores of the MJ2, hkMJ2, and LP groups were 2.6 ± 0.3, 3.8 ± 0.1, and 2.7 ± 0.1, respectively, which means that insulin resistance in the bacteria-treated groups showed a significant decrease compared with that in the model group. The results suggest that the administration of MJ2 and hkMJ2 alleviates insulin resistance in HFD-induced obese mice. In particular, MJ2 reduced blood glucose and fasting blood insulin more effectively than hkMJ2.

### MJ2 and hkMJ2 ameliorated liver damage induced by HFD intake in mice

**Figure 8:**
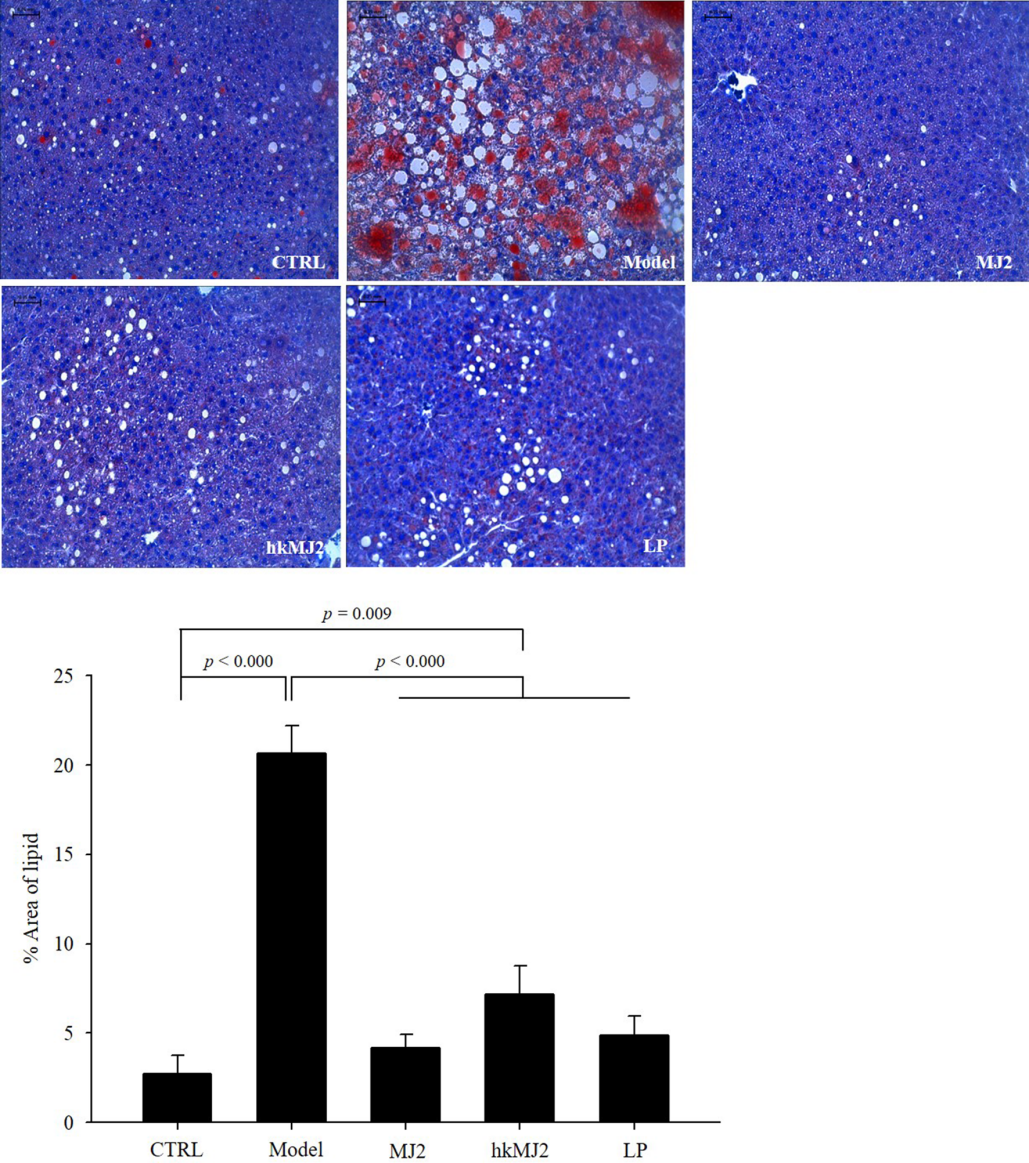
Effect of MJ2 and hkMJ2 on lipid accumulation in the livers of HFD-induced obese mice (n = 6/group). The lipid accumulation in the liver was investigated at least 4 parts randomly selected per each sample by staining with oil red O. The scale bars indicate 0.25 mm, and the slides were observed at 200× magnification. The data indicate the mean ± SD, and the* p* values were determined by ANOVA and Tukey’s HSD test.

The changes in the conditions related to lipid metabolism by obesity cause an accumulation of lipids in hepatocytes. To examine the alleviating effect of MJ2 and hkMJ2 on liver damage, lipid accumulation in the liver and serum biomarkers related to hepatotoxicity were investigated. The liver tissues were stained with H&E to observe lipid accumulation. The accumulated lipids in the observed liver tissues significantly increased 7.6-fold in the model group compared with the CTRL (Fig. 8). However, the MJ2, hkMJ2, and LP groups had significantly decreased lipid accumulation compared with that in the model group. The % areas of lipids decreased 5-, 2.9-, and 4.3-fold in the MJ2, hkMJ2, and LP groups, respectively, compared with the model group. To examine liver function and damage, glutamic oxaloacetic transaminase (GOT) and glutamic pyruvic transaminase (GPT) were measured. The GOT and GPT levels in serum were significantly increased in the model group compared with the CTRL (Supplementary Fig. 4). The serum GOT and GPT levels were significantly reduced by treatment with MJ2 and hkMJ2. The results suggest that oral administration of MJ2, hkMJ2, and LP effectively ameliorated liver damage in HFD-induced obese mice. In particular, the MJ2 group showed the greatest liver protecting effect compared with the hkMJ2 and LP groups.

## Discussion

The main effects of probiotics have been focused on protection against pathogens, immunomodulation, balancing gut microbiota, and the maintenance of gut barrier integrity^23^. When *Lactobacillus paracasei*, a typical probiotic, monocolonizes germ-free mice, it upregulates angiopoietin-like 4 protein, which contributes to decreasing fat mass, in an HFD-fed mouse model^24^. In diet-induced obese mice, the oral administration of *L. plantarum* alleviates metabolic syndromes caused by obesity^25,26^. The potential probiotic *P. freudenreichii* MJ2 could suppress lipid accumulation and alleviate obesity and metabolic disorders related to obesity, including hyperlipidemia, type 2 diabetes mellitus, and liver damage, in HFD-induced obese mice. The surface layer protein SlpB of *P. freudenreichii* reduces mucositis^10^ and is involved in adhesion to human intestinal HT-29 cells, which explains why the *P. freudenreichii* S-layer protein might be the determinant of its probiotic action^11^. The extracellular vesicles of *P. freudenreichii* inhibit inflammatory activity through NF-κB pathway modulation^27^. In this study, we focused on metabolic modulation through the probiotic action of *P. freudenreichii. P. freudenreichii* MJ2 effectively inhibited lipid accumulation and adipocyte differentiation, and the antiobesity effect of *P. freudenreichii* MJ2 was proven to be superior to that of *L. plantarum*, which is known as an antiobesity probiotic bacterium. *L. plantarum* shows improvement effects on lipid metabolism disorder in HFD-fed mice by modulating gut microbiota^28^. The antioxidant makers decreased in HFD-fed mice and *L. plantarum* attenuated oxidative stress by increase of antioxidant markers (catalase, superoxide dismutase, and reduced glutathione) in the serum of HFD-fed mice^29^, which suggests that antioxidant activity of *L. plantarum* may contribute to improve HFD-induced obesity.

Probiotics are an excellent dietary supplement for ameliorating host morbidity, including obesity, but there are also safety issues that make probiotics inappropriate for vulnerable hosts^30,31^. The administration of live organisms may cause bacteremia and the acquisition or transmission of antibiotic resistance in vulnerable hosts. To prevent these problems, the cure effects of heat-killed bacteria or inactivated probiotics have been studied to treat pathological conditions and imbalances of gut microbiota composition without the risks related to the translocation of bacteria and acquisition of antibiotic resistance in vulnerable patients^32^. In the case of heat-killed bacteria, cell walls are broken down, and cell wall components such as lipoteichoic acids, peptidoglycans, surface layer protein, and cytoplasmic contents are released. Released bacterial components might act as metabolic modulators that interact with the intestinal epithelium, dendritic cells, and other immune cells of the host by Toll-like/signal transduction receptors^33^. In this study, live and heat-killed *P. freudenreichii* MJ2 showed similar antiobesity effects. The results suggested that certain component(s) of *P. freudenreichii* MJ2 that are not inactivated by heat might ameliorate obesity. This is a particularly important advantage of developing *P. freudenreichii* MJ2 as an agent (drug or functional food) to treat obesity.

Although both live and heat-killed *P. freudenreichii* MJ2 improved obesity in HFD-induced obese mice, there are some differences in detail. ATGL involved in lipolysis significantly increased in the MJ2 compared with the hkMJ2. In addition, the size of adipocytes in eWAT in the MJ2 significantly smaller than that in the hkMJ2. The results suggested that live *P. freudenreichii* MJ2 was more effective in decrease of adipocyte size and in increase of lipolysis than the hkMJ2, which might result that the MJ2 lost more weight than the hkMJ2. In addition, fasting blood insulin and insulin resistance in the MJ2 significantly low compared with the hkMJ2, which suggested that live *P. freudenreichii* MJ2 might be more effective in improving diabetes. It is assumed that the active component(s) of *P. freudenreichii* MJ2 for antiobesity activity might be not a single substance because both live and heat-killed *P. freudenreichii* MJ2 show antiobesity activity, however, live *P. freudenreichii* MJ2 is more effective than heat-killed *P. freudenreichii* MJ2. We need to identify the active component(s) of *P. freudenreichii* MJ2 that play a role in the amelioration of obesity.

Adipose tissue is divided into white adipose tissue (WAT) and brown adipose tissue (BAT). WAT is specialized to retain lipids as an energy source, while BAT includes pigmented mitochondria and is supplied sufficient blood to consume energy by nonshivering thermogenesis^18^. WAT correlates with obesity and metabolic diseases caused by obesity. In this study, the expression levels of adipogenic factors (PPARγ and C/EBPα) and lipogenic factors (FAS, SCD-1, and ACC) in eWAT of HFD-induced obese mice were inhibited by MJ2 and hkMJ2, which means that *P. freudenreichii* MJ2 might prevent lipid accumulation in adipocytes. Adipogenesis, lipogenesis, and lipolysis consistently occur as part of the regulation of the metabolic cycle^34^. In the lipolytically active state, ATGL hydrolyzes triacylglycerol in lipid droplets to diacylglycerol and fatty acids, and HSL hydrolyzes diacylglycerol to monoacylglycerol and fatty acids^35^. MJ2 and hkMJ2 increase the expression levels of lipolytic factors (ATGL and HSL). Thus, MJ2 and hkMJ2 inhibit the expression of adipogenic and lipogenic factors and stimulate the expression of lipolytic enzymes, which means that *P. freudenreichii* MJ2 reduces fat accumulation, ameliorating obesity regardless of whether the bacterium is live or heat-killed. Although metabolic disorders such as dyslipidemia, insulin resistance, and inflammation do not necessarily relate to adipocyte size, increased adipocyte size, including lipid overflow, might predispose the organism to diverse negative metabolic changes when adipocyte size exceeds a certain threshold^36^. *P. freudenreichii* MJ2 prevents increases in adipocyte size, which might prevent adipose tissue expandability, ameliorating diverse negative metabolic changes induced by lipid accumulation.

Type 2 diabetes, an obesity-related metabolic disease, shows a different pathophysiology from type 1 diabetes and is induced by insulin resistance. The main causes of type 2 diabetes are dysfunction of pancreatic β-cells causing a reduction in the production of insulin or an increase in peripheral insulin resistance^37^. In the HFD-induced obese mouse model, the body weight gain induced by HFD feeding may cause insulin resistance, and the nonesterified fatty acids released from adipose tissue in obesity and both insulin resistance and dysfunction of pancreatic β-cells may be linked. HOMA-IR means homeostatic model assessment of insulin resistance, and the optimal range is from 0.5 to 1.4. HOMA-IR scores greater than 1.9 or 2.9 indicate early or significant insulin resistance, respectively^38^. In this study, the model group showed significant insulin resistance following an increase in the HOMA-IR index. However, increased glucose and fasting blood insulin were significantly reduced by oral administration of *P. freudenreichii* MJ2. These results suggest that *P. freudenreichii* MJ2 alleviates type 2 diabetes induced by HFD feeding, which means that *P. freudenreichii* MJ2 might act as a modulator of lipid metabolism as well as glucose metabolism.

NAFLD, typically caused by obesity, is an early step in the progression of liver diseases. It includes simple steatosis, which accelerates de novo lipogenesis, and nonalcoholic steatohepatitis, which is accompanied by inflammation and fibrosis. The liver gradually becomes hypertrophic as fat continues to accumulate in the liver^39^. HFD can easily lead to NAFLD, which progresses to abnormal accumulation of fat in the liver. The changed conditions related to lipid metabolism caused by obesity produce an accumulation of lipids in hepatocytes. Accumulated lipids in the liver induce the infiltration of immune cells into the liver and consequent inflammation. The repetition of these processes leads to fibrosis of the liver^40,41^. Damage to liver tissue can be observed by not only the amount of accumulated fat but also morphological features. Clear vacuoles containing lipids are usually observed in liver sections with severe steatosis^42^. Biomarkers (GOT and GTP) for liver damage in HFD-induced obese mice are improved by treatment with MJ2 and hkMJ2. In addition, MJ2 and hkMJ2 decreased lipid vacuoles in the liver, which means that *P. freudenreichii* MJ2 might improve liver damage such as steatosis.

In conclusion, obesity and metabolic disorders related to obesity, such as diabetes, hyperlipidemia, and NALFD, are rapidly increasing with Western eating habits and are becoming more serious. A potential probiotic isolated from raw milk, *P. freudenreichii* MJ2, not only shows an antiobesity effect by inhibiting lipid accumulation and adipocyte differentiation in a 3T3-L1 preadipocyte and HFD-induced obese mouse model but also improves metabolic disorders in HFD feeding conditions. It reduces blood cholesterol and prevents liver damage. In addition, *P. freudenreichii* MJ2 effectively alleviates diabetes by reducing blood glucose and fasting blood insulin. Therefore, *P. freudenreichii* MJ2 is a potential probiotic bacterium that improves obesity by modulating lipid metabolism.

## Materials and methods

### Materials

Reinforced clostridial medium (RCM) and De Man, Rogosa and Sharpe (MRS) medium were purchased from Oxoid Ltd. (Hampshire, United Kingdom) and BD Biosciences (Bergen County, NJ, USA), respectively. Dulbecco’s modified Eagle’s medium (DMEM), bovine calf serum (BCS), penicillin/streptomycin (P/S), and trypsin-EDTA were purchased from HyClone (Logan, UT, USA). 3-Isobutyl-1-methylxanthine (IBMX), dexamethasone, and insulin were obtained from Sigma-Aldrich (St. Louis, MO, USA). Dimethyl sulfoxide (DMSO) was purchased from DAEJUNG (Siheung, Korea).

### Bacterial strains and culture conditions

*Propionibacterium freudenreichii* MJ2 was isolated from raw milk and identified through 16S rDNA sequencing. *Lactobacillus plantarum* KACC15357 was purchased from the Korean Agricultural Culture Collection (KACC) (Wanju, Jeollabuk-do, Korea). *P. freudenreichii* MJ2 was cultured in RCM broth under anaerobic conditions at 30 ℃ for 48 h when the bacterium reached stationary phase, and *L. plantarum* was cultured in MRS broth under anaerobic conditions at 37 ℃ for 24 h when the bacterium reached stationary phase. After cultivation, cells were collected by centrifugation at 8000×*g* for 10 min at 4 ℃ and then washed twice with PBS. The washed pellet was resuspended in PBS and the cells were counted using a Neubauer-improved counting chamber (superior Marienfeld, Germany). After adjusting the concentrations of cells to 10^6^, 10^7^, and 10^8^ cells/mL, the cells were heat-killed at 80 ℃ for 30 min to prepare heat-killed bacteria.

### 3T3-L1 cell culture and differentiation

3T3-L1 preadipocytes (CL-173) were obtained from the American Type Culture Collection (ATCC) (Manassas, VA, USA). The cells were cultured in DMEM containing 10% BCS and 1% P/S at 37 ℃ in a humidified 5% CO_2_ atmosphere, and the medium was changed every two days^43^. For differentiation, cells were maintained at 100% confluence for two days. After two days, the culture medium was changed to differentiation induction medium (MDI medium: DMEM containing 10% FBS, 1% P/S, 0.5 mM IBMX, 1 μM dexamethasone, and 5 μg/mL insulin). The day the medium was replaced with MDI medium was day 0. On day 2, the medium was changed to insulin medium, which was DMEM containing 5 μg/mL insulin. The medium was then replaced with new insulin medium every two days until day 8. Cells were treated with heat-killed *P. freudenreichii* MJ2 (hkMJ2) (10^6^, 10^7^, and 10^8^ cells/mL) and heat-killed LP (hkLP) (10^7^ and 10^8^ cells/mL) during the differentiation period (days 0 ‒ 8). The scheme for the differentiation of preadipocytes into adipocytes is shown in Supplementary Fig. 5.

### Oil red O staining

The accumulation of lipids in differentiated 3T3-L1 adipocytes was measured by quantifying oil red O staining. On day 8, the cells were stained with oil red O, washed twice with PBS, and then fixed with 10% formalin for 1 h at room temperature as described previously^44^. Fixed cells were washed twice with distilled water and incubated with 60% isopropanol for 5 min. Incubated cells were soaked in oil red O working solution for 30 min at room temperature. After staining, the cells were washed with distilled water four times and dried completely. The oil red O stain was collected with 100% isopropanol, and the optical density (OD) was measured at 500 nm using a microplate reader (SpectraMax 340PC). The relative lipid accumulation (%) was obtained by comparing the OD value of the differential control to the OD value of each treated cell.

### Experimental animals and HFD-induced obesity

Four-week-old male C57BL/6N mice (14‒16 g) were obtained from Kotech (Pyeongtaek, Korea). The protocols of this study were managed according to an institutionally approved protocol from the Korea University Institutional Animal Care and Use Committee (Approval No. KUIACUC-2018-0007). All experimental procedures were in accordance with the Guide for the Care and Use of Laboratory Animals (NIH Publication No. 85-23, 1996). The mice (3–4 mice/cage) were raised at a temperature of 22 ± 1 ℃, intensity of illumination of 200 ± 50 lx under a 12 h light–dark cycle, and relative humidity of 50 ± 5%. All the mice had free access to food (Supplementary Table 3) and water. After acclimation for one week, mice were randomly separated into 5 groups (n = 6/group). Group 1 (the normal control; CTRL) was treated with vehicle (PBS) and a normal-fat diet; group 2 (the HFD-induced obesity model; Model) was treated with vehicle and HFD; group 3 (MJ2) was treated with live MJ2 (10^8^ cfu/day) and HFD; group 4 (hkMJ2) was treated with heat-killed MJ2 (10^8^ cells/day) and HFD; and group 5 (the positive control; LP) was treated with live *Lactobacillus plantarum* (10^8^ cfu/day) and HFD. To induce obesity, HFD was fed to all groups except the normal control group without any treatment until the average weight of the HFD-fed groups increased by 25% compared with the CTRL (6 weeks). After that, vehicle (200 μL of PBS) and MJ2, hkMJ2, and LP (10^8^ cells in 200 μL of PBS) were orally administered daily for 8 weeks. The scheme to produce HFD-induced obese mice is shown in Supplementary Fig. 6.

### Blood and tissue preparation

The mice were fasted for 12 h and sacrificed through cardiac puncture while anesthetized by breathing isoflurane. Collected blood was centrifuged for 10 min at 3000×*g* to separate serum. The livers and epididymal white adipose tissues (eWAT) were dissected intact, washed with PBS immediately, and weighed. The relative weight (g) of tissues was calculated as the weight of tissue/the body weight. Organ samples were stored at − 80 ℃ until analysis.

### Blood glucose and insulin analysis

Blood glucose concentrations were measured as soon as blood was collected using Accu-Chek Guide (Roche, Basel, Switzerland). The insulin level in mouse serum was determined by using an Ultrasensitive Mouse Insulin ELISA Kit (catalog #90080; Crystal Chem, Downers Grave, IL, USA) according to the manufacturer’s instructions. Insulin resistance was calculated by applying homeostasis model assessment (HOMA-IR) using the HOMA2 calculator (Diabetes Trials Unit; DTU, Oxford, UK).

### Histological analysis

Dissected liver and eWAT were subjected to formalin fixation with 4% formaldehyde immediately after excision. The fixed liver was quickly frozen with liquid nitrogen and sectioned at approximately 3‒5 μm thickness. The eWAT slides were soaked in absolute propylene glycol for 5 min, and then the tissues were stained with oil red O solution for 30 min at room temperature. The nuclei were stained with Mayer’s hematoxylin solution. The stained slides were washed three times with water. Fixed eWAT was dehydrated using diverse concentrated ethanol (80‒100%) and cleaned with xylene to embed the tissues in paraffin. The embedded tissues were sectioned at approximately 3‒5 μm thickness and subjected to H&E staining. The stained liver tissues and eWAT slides were observed by using a phase contrast microscope (Leica DM750, Leica Microsystems, Wetzlar, Germany) to detect lipid droplets in liver tissues and compare the size of adipocytes in eWAT. The area of lipid droplets stained with oil red O solution was determined with ImageJ software (National Institutes of Health, Bethesda, MD, USA). The sizes of adipocytes were also measured using ImageJ software according to a previously described method^45^.

### Quantitative real-time polymerase chain reaction (qPCR)

Total RNA was extracted using TRIzol reagent (Thermo Fisher Scientific, Waltham, MA, USA) following the manufacturer’s instructions. The total concentration of extracted RNA was measured using a NanoDrop (ND-1000 spectrophotometer, Thermo Fisher Scientific, Waltham, MA, USA). After quantification of RNA, the RNA was converted into cDNA using a revertAid First Stand cDNA Synthesis Kit (Thermo Fisher Scientific). The cDNA was amplified by qPCR with a KAPA SYBR FAST qPCR Kit (KAPA Biosystems, Wilmington, NC, USA) by using QuantStudio 6 Flex (Life Technologies, Carlsbad, CA, USA). The primer sequences used in this study are shown in Supplementary Table 4. The primers were purchased from Bioneer (Seoul, Korea). The loaded cDNA with qPCR reaction reagents was preheated to 95 ℃ followed by 40 cycles at 95 ℃ for 15 s, 60 ℃ for 15 s, and 72 ℃ for 30 s. The relative mRNA expression was assessed based on the number of cycles that reached a specific detection threshold (Ct value). The resulting Ct values were calculated by a comparative Ct method (ΔΔCt method)^46^. ATP synthase mitochondrial F1 complex assembly factor 1 (*ATPF-1*) was used as a reference gene.

### Western blot analysis

Protein extraction was performed from eWAT (50 mg) using a Taco prep bead beater with RIPA buffer (50 mM Tris-HCl pH 7.5, 150 mM sodium chloride, 2 mM EDTA, 1% sodium deoxycholate, 1% Triton X-100 and 0.1% sodium dodecyl sulfate) and incubated at 4 ℃ for 30 min to induce cell lysis. The lysed tissues were centrifuged at 13,600×*g* at 4 ℃ for 20 min. The concentration of extracted protein was determined using a BCA assay (Thermo Scientific). Bovine serum albumin (BSA) (Bio-Rad, Hercules, CA, USA) was measured as a standard for quantification. Equally quantified protein (45 μg) was separated by 10% sodium dodecyl sulfate–polyacrylamide gel electrophoresis (SDS-PAGE). The separated proteins on the gel were transferred to a polyvinylidene difluoride (PVDF) membrane (Millipore) using a Trans-Blot semidry transfer cell (Bio-Rad). The PVDF membranes were soaked in 6% nonfat skimmed milk (Neogen, Lansing, MI, USA) in PBS-T (PBS with 0.05% Tween 20) for 1 h at room temperature and then washed with PBS-T for 10 min at room temperature three times. The washed PVDF membranes were reacted with primary antibodies overnight at 4 ℃. GAPDH antibody (GTX100118, GeneTex, Irvine, CA, USA; 1:1000 dilution) was used as an endogenous control. Anti-PPARγ (GTX57756, GeneTex; 1:1000 dilution), anti-C/EBPα (MA1825, Thermo Scientific; 1:500 dilution), anti-FAS (PA522061, Thermo Scientific; 1:1000 dilution), anti-SCD-1 (MA514885, Thermo Scientific; 1:1000 dilution), anti-ACC (MA515025, Thermo Scientific; 1:500 dilution), anti-pACC (PA517725, Thermo Scientific; 1:500 dilution), anti-ATGL (MA514990, Thermo Scientific; 1:1000 dilution), anti-HSL (PA517196, Thermo Scientific; 1:1000 dilution), and anti-pHSL (PA517488, Thermo Scientific; 1:1000 dilution) were used as primary antibodies. After reaction with primary antibodies, the membranes were washed with PBS-T for 10 min three times and reacted with the secondary antibody for 1 h at room temperature. Rabbit IgG antibody (HRP) (GTX213110-01, GeneTex; 1:2000 dilution) was used as a secondary antibody. The membranes were washed with PBS-T again, and the bands were detected using a SuperSignal West Femto Maximum Sensitivity Substrate Kit (Thermo Scientific) and captured by FluorChem E (Proteinsimple, San Jose, CA, USA). The protein bands were quantified using ImageJ software (Softomic, Barcelona, Spain).

### Statistical analysis

All evaluations were performed using Statistical Package for the Social Science (SPSS) statistical analysis program Ver. 25. The results were evaluated with a one-way analysis of variance (ANOVA), and a post hoc test was conducted using Tukey’s honest significant difference (HSD) method to confirm significant differences between the groups. Data are indicated as the mean ± standard deviation (SD). A *p *value of < 0.05 was considered significant.

## Supplementary Information

Supplementary Information.
