# Supplementary material for: Antiobesity and antidiabetic effects of the dairy bacterium Propionibacterium freudenreichii MJ2 in high-fat diet-induced obese mice by modulating lipid metabolism
Source: Sci Rep. 2021 Jan 28;11:2481. doi: 10.1038/s41598-021-82282-5 (PMC7844274; doi:10.1038/s41598-021-82282-5)
Supplement: Supplementary file 1 — Supplementary Information. [file 41598_2021_82282_MOESM1_ESM.pdf]

Antiobesity and antidiabetic effects of the dairy bacterium *Propionibacterium freudenreichii* MJ2 in high-fat diet-induced obese mice by modulating lipid metabolism

Authors:

Mirae An[1,2,3], Yeon-Hee Park[3] & Young-Hee Lim[2,4,5*]

Affiliations:

[1] Department of Healthcare Sciences, Graduate school, Korea University, Seoul 02841, Republic of Korea.

[2] BK21FOUR R&E Center for Learning Health Systems, Korea University, Seoul 02841, Republic of Korea.

[3] Department of Public Health Sciences, Graduate school, Korea University, Seoul 02841, Republic of Korea.

[4] Department of Integrated Biomedical and Life Sciences, Graduate School, Korea University, Seoul 02841, Republic of Korea.

[5] Department of Laboratory Medicine, Korea University Guro Hospital, Seoul 08308, Republic of Korea

Methods

Cell viability. 3-[4,5-Dimethylthiazol-2-yl]-2,5-diphenyltetrazolium bromide (MTT, Amresco, Solon, OH, USA) assay was carried out to determine the effect of MJ2 on cell viability. 3T3-L1 pre-adipocytes (5×10^4 cells/mL) were seeded in a 96-well plate and cultured at 37 °C in humidified 5% CO_2 atmosphere for 24 h. Then the medium was changed from DMEM containing 10% FBS to DMEM without FBS and treated with heat-killed *P. freudenreichii* MJ2 (hkMJ2) by concentration (10^5, 10^6, 10^7, and 10^8 cells/mL). The negative control was treated PBS. After incubating for 24 h, the medium was switched to the medium containing 0.125 mg/mL of MTT and then cells were incubated for 1 h in the dark condition. The medium was completely removed and 200 μL of DMSO was added into each well. After 30 min, the absorbance of each well was measured using a microplate reader (SpectraMax 340PC, Molecular devices, CA, USA) at 540 nm. The relative cell viability (%) was calculated by comparing OD value of the negative control to OD value of each treated cells.

Blood biochemical analysis. Separated serum was analyzed some biochemical markers using FUJI DRY-CHEM slides (FUJIFILM Co. Tokyo, Japan). Glutamic oxaloacetic transaminase (GOT) and glutamic pyruvic transaminase (GPT) level were measured to examine hepatotoxicity and liver damage. Also, total cholesterol (TCHO), triglycerides (TG), and high-density lipoprotein cholesterol (HDL) level were analyzed. Low-density lipoprotein cholesterol (LDL) was calculated by applying Frieldwann's equation (1):

$$\mathrm{LDL} = \left[\mathrm{TC} - \left\{\mathrm{HDL} + \left(\frac{\mathrm{TG}}{5}\right)\right\}\right] \quad (1)$$

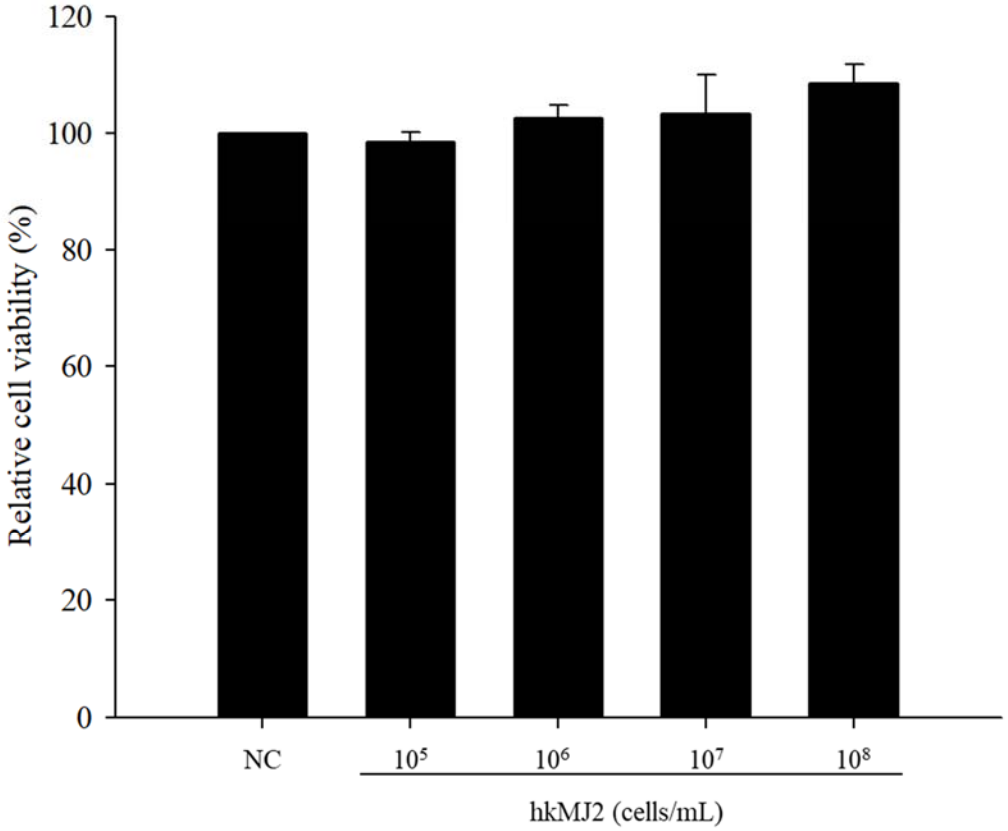

Supplementary Fig. 1. Relative viability of heat-killed *P. freudenreichii* MJ2 (hkMJ2)-treated 3T3-L1 pre-adipocytes. 3T3-L1 pre-adipocytes were treated with various concentrations (10^5, 10^6, 10^7, and 10^8 cells/mL) of hkMJ2 for 24 h. The data indicate the mean ± SD of three independent experiments.

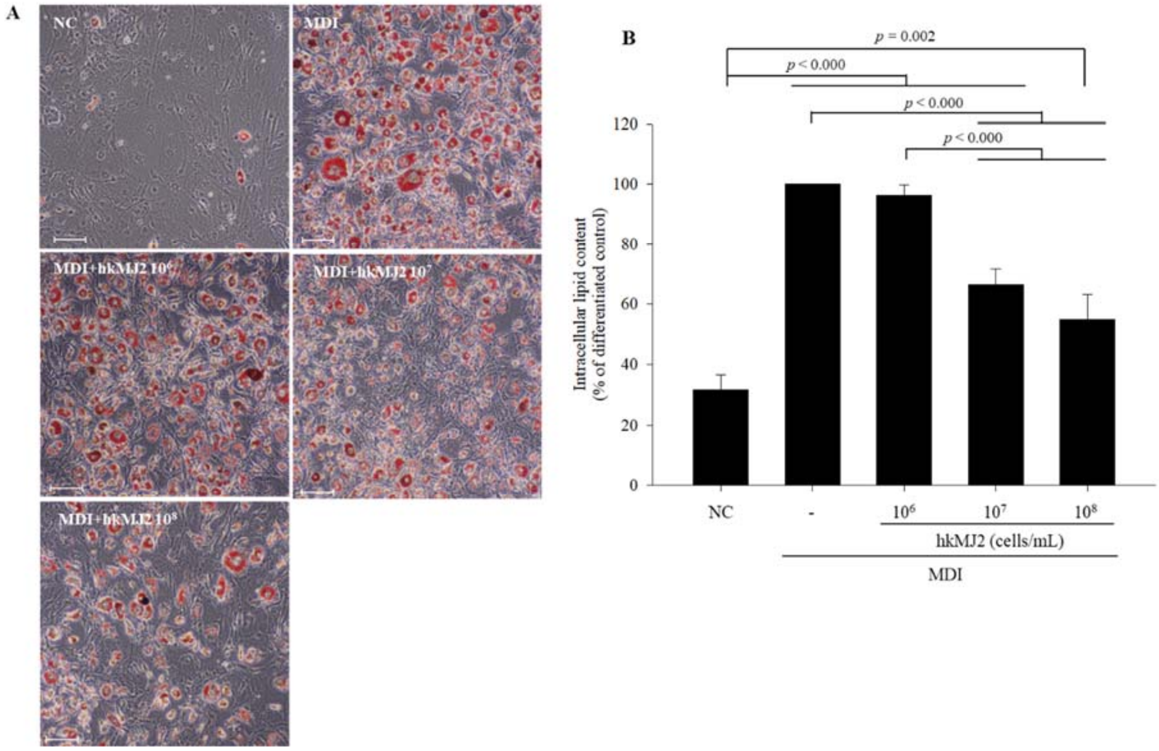

Supplementary Fig. 2. Inhibitory effects of heat-killed *P. freudenreichii* MJ2 on lipid accumulation in 3T3-L1 adipocytes. 3T3-L1 preadipocytes were differentiated by treatment with differentiation-inducing medium containing MDI (IBMX + dexamethasone + insulin), and hkMJ2 were simultaneously administered at the designated concentrations during the period of differentiation. (A) Lipid droplets of the differentiated cells were visualized by oil red O staining (100× magnification) and the scale bars indicate 0.5 mm. (B) Lipid accumulation was quantified by measuring oil droplets stained with oil red O and comparing them with the cells treated with MDI alone. The data indicate the mean ± SD of three independent experiments. The p values are determined by ANOVA and Tukey's HSD test.

GAPDH

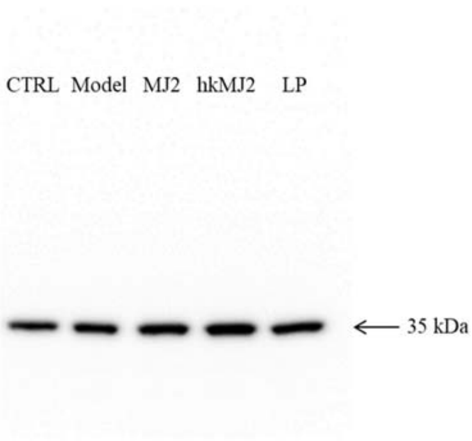

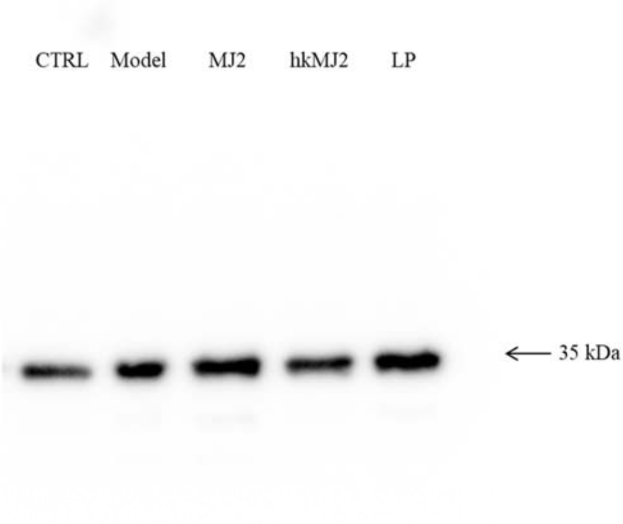

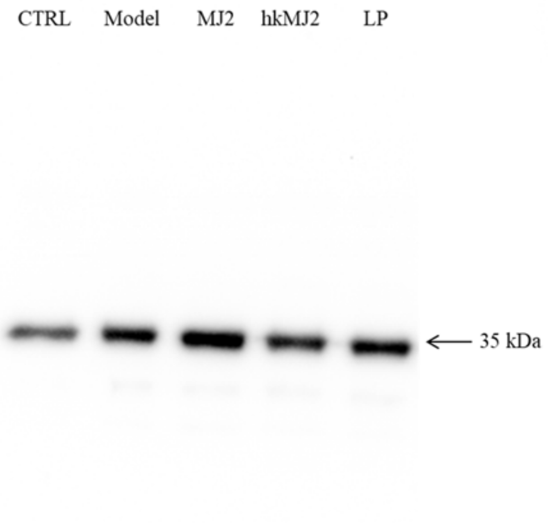

PPARγ

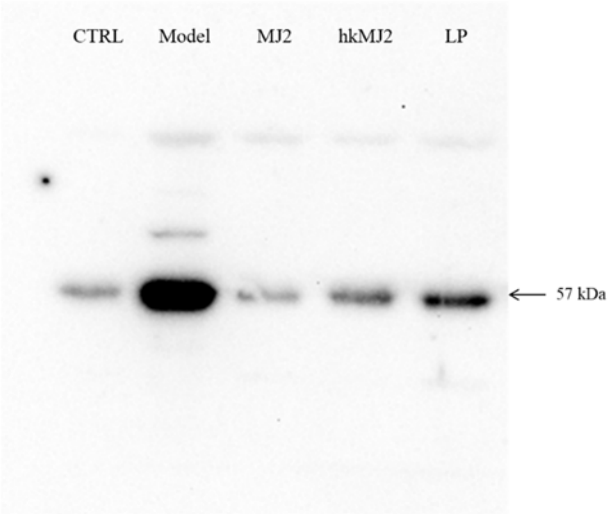

C/EBPα

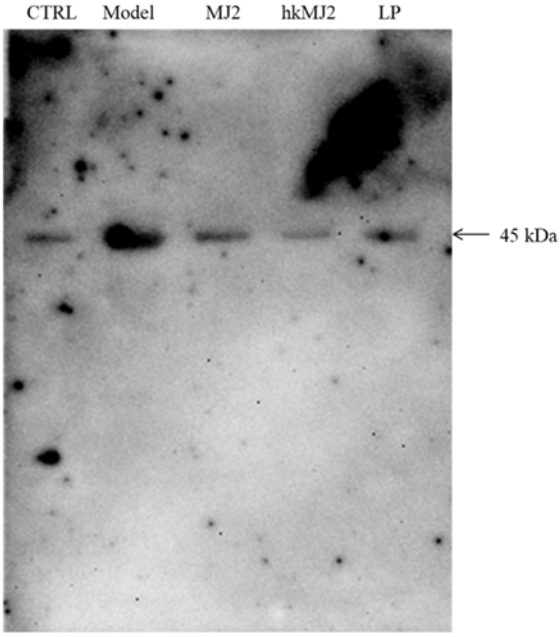

FAS

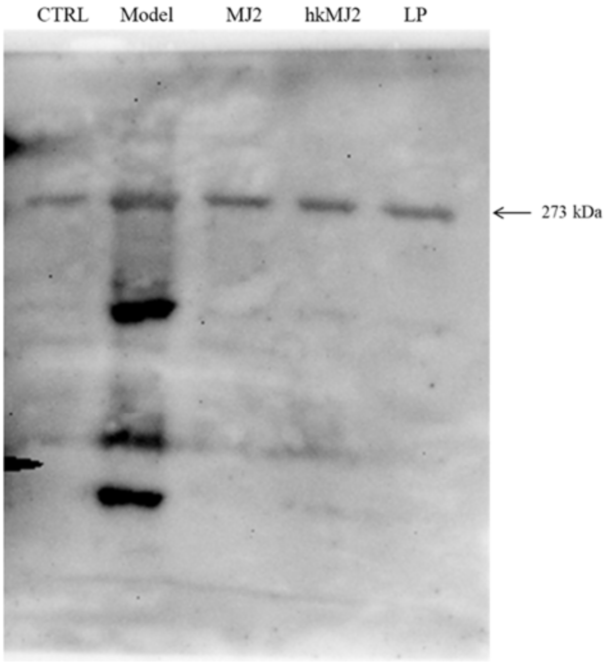

SCD1

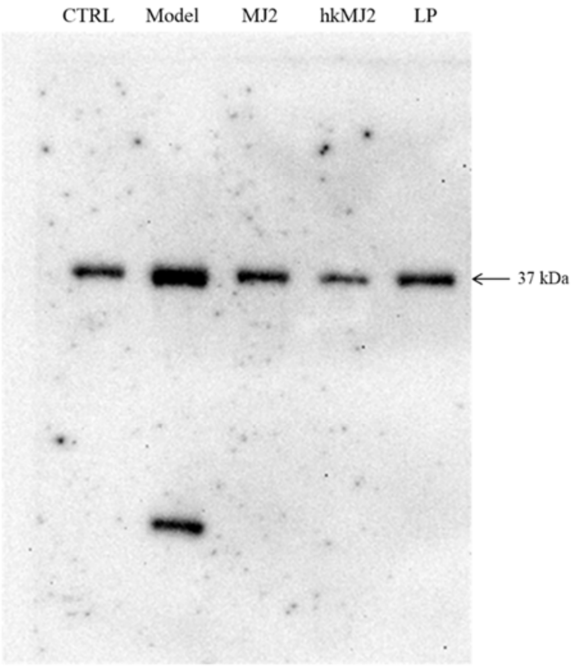

ACC

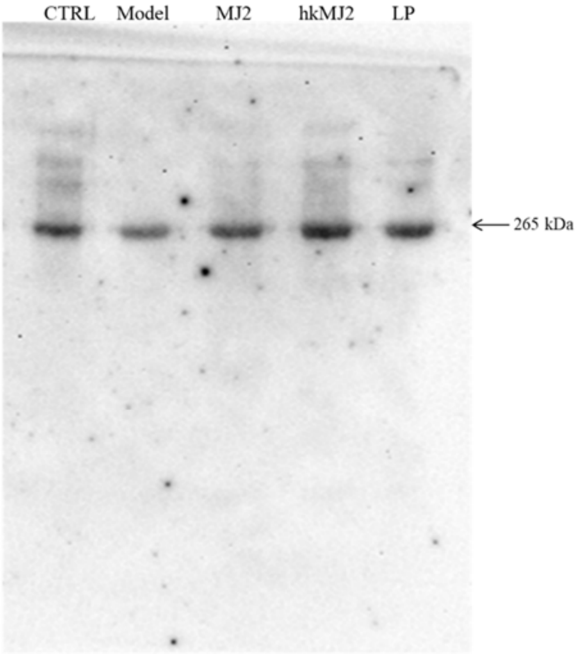

pACC

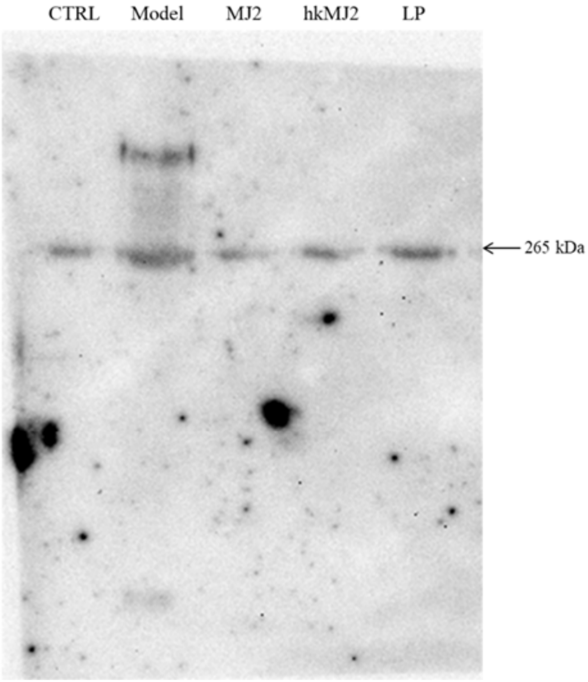

ATGL

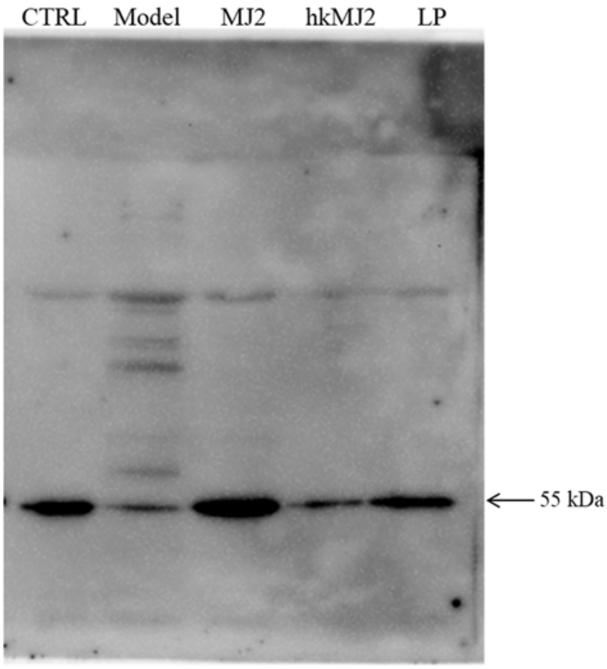

HSL

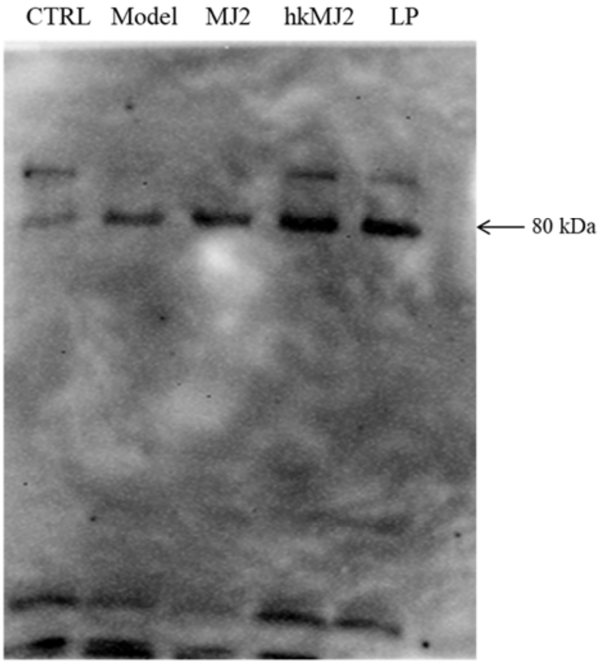

pHSL

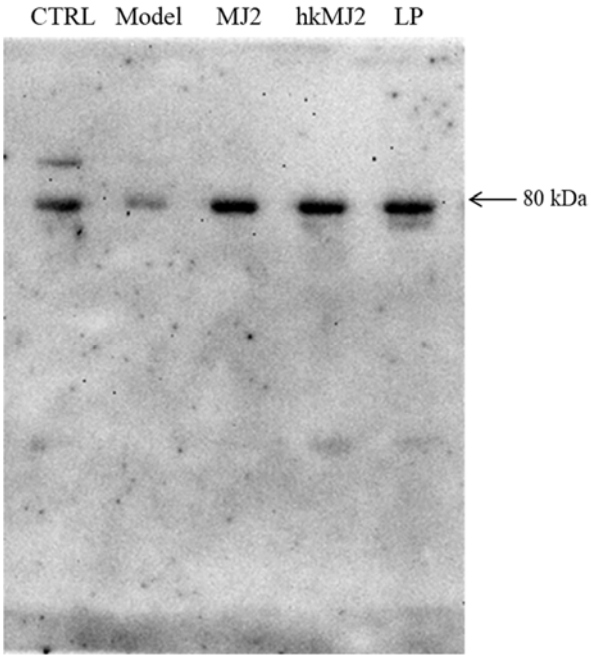

Supplementary Fig. 3. The full-length blots of the proteins shown in Fig. 5.

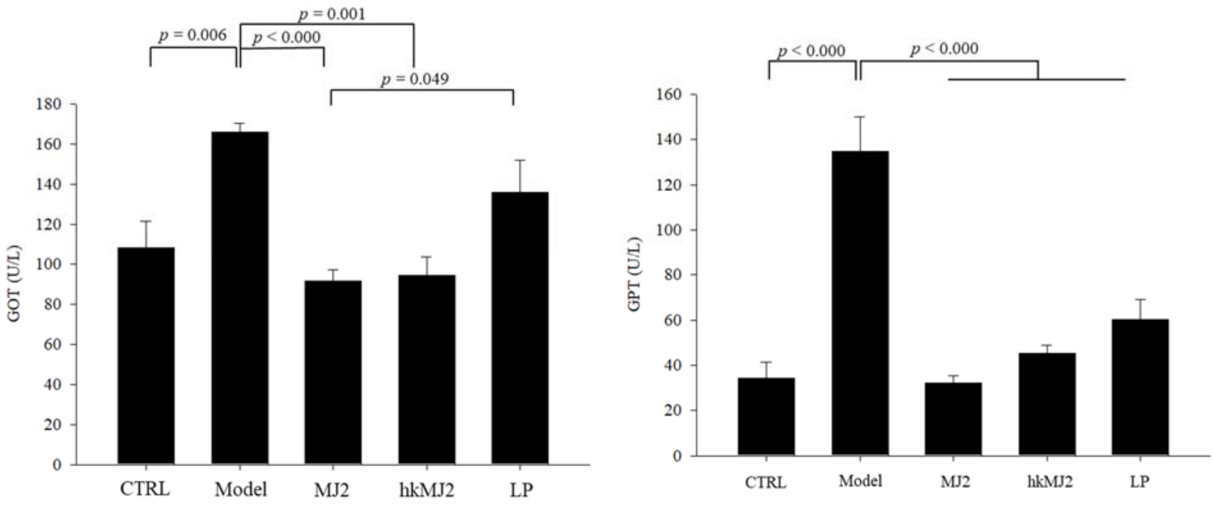

Supplementary Fig. 4. The levels of serum biomarkers related to hepatotoxicity. The data indicate the mean ± SD and the *p* values are determined by ANOVA and Tukey's HSD test.

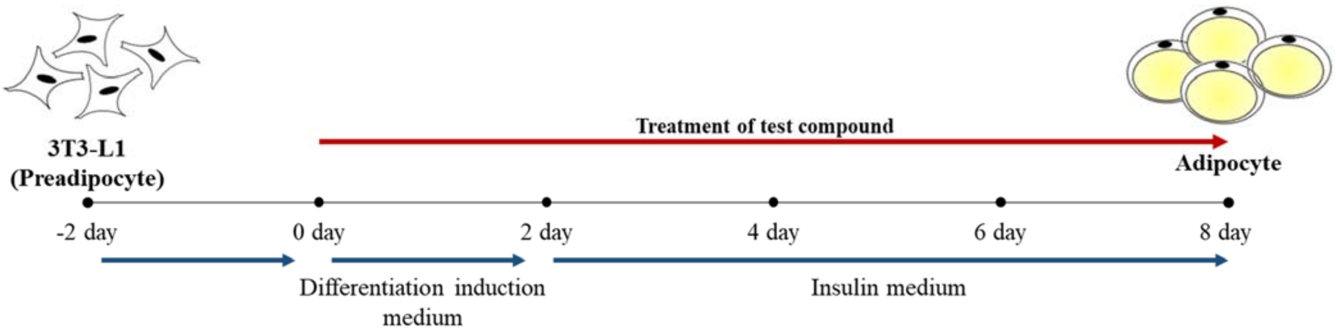

Test compound	Differentiation
Negative control (PBS)	×
MDI	O
MDI + heat-killed *P. freudenreichii* MJ2 10^7 cells/mL	O
MDI + heat-killed *P. freudenreichii* MJ2 10^8 cells/mL	O
MDI + heat-killed *L. plantarum* 10^7 cells/mL	O
MDI + heat-killed *L. plantarum* 10^8 cells/mL	O

MDI (IBMX + Dexamethasone + Insulin)

Supplementary Fig. 5. The scheme for differentiation of pre-adipocyte into adipocyte.

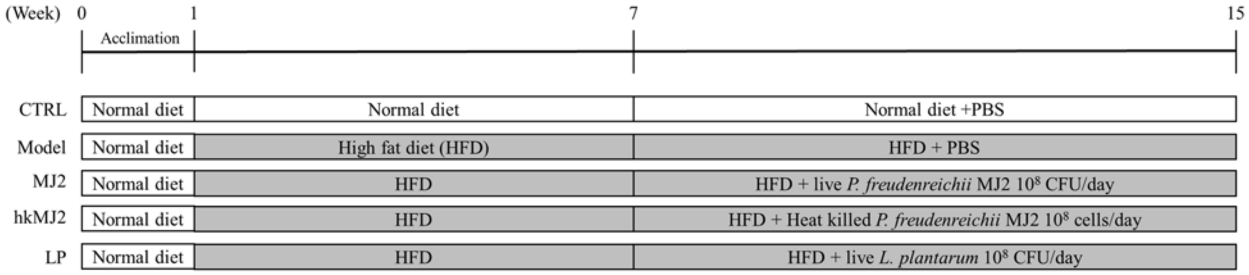

Supplementary Fig. 6. The scheme to produce HFD-induced obese mice.

Supplementary Table 1. Effect of live and heat-killed *P. freudenreichii* on body weight change and food efficiency ratio in HFD-induced obese mice.

Group	Body weight (g)		Body weight gain (g)	Food intake (g/day)	Food efficiency ratio
	Initial	Final			
CTRL	14.9 ± 0.6	30.3 ± 2.2[a]	15.3 ± 2.0[a]	2.4 ± 0.2	6.5 ± 0.9[a]
Model	15.2 ± 0.7	43.7 ± 4.8[b]	28.5 ± 4.5[b]	2.2 ± 0.1	12.7 ± 2.3[b]
MJ2	15.0 ± 0.7	34.8 ± 2.4[a,c]	19.8 ± 2.5[a,c]	2.3 ± 0.2	8.5 ± 1.1[a,c]
hkMJ2	14.7 ± 0.5	36.8 ± 2.2[c]	22.1 ± 2.6[c]	2.2 ± 0.2	10.0 ± 1.1[c]
LP	15.1 ± 0.5	38.5 ± 3.4[b,c]	23.4 ± 3.1[c]	2.3 ± 0.1	10.0 ± 1.3[c]

The data indicate the mean ± SD and one-way ANOVA/Tukey's HSD analysis was performed and different letters in the same column indicate significant difference ($p < 0.05$).

Supplementary Table 2. Effects of MJ2 and hkMJ2 on the blood cholesterol.

Group	(mg/dL)			
	**TCHO**	**TG**	**HDL**	**LDL**
CTRL	135.3 ± 32.9^{a}	63.8 ± 13.6	62.8 ± 14^{a}	58.9 ± 17.7^{a}
Model	193.8 ± 31.7^{b}	59.5 ± 15.5	90.2 ± 11.4^{b}	91.8 ± 22.8^{b}
MJ2	137.7 ± 30.5^{a}	53.5 ± 10.5	65.2 ± 14.6^{a}	61.8 ± 15.8^{a}
hkMJ2	158 ± 20.9^{a,b}	51.2 ± 6.4	73.5 ± 10.5^{a,,b}	74.3 ± 11.0^{a,b}
LP	157.2 ± 28.8^{a,b}	59.8 ± 7.7	73.5 ± 17.5^{a,,b}	71.9 ± 11.8^{a,b}

The data indicate the mean ± SD. Data sharing the same letter are not significantly different ($p < 0.05$). One-way ANOVA/Tukey HSD analysis was performed.

Supplementary Table 3. Compositions of experimental diets used in the study.

Component	Normal fat diet		High-fat diet	
	g %	kcal %	g %	kcal %
Protein	19.2	20	26.2	20
Carbohydrate	67.3	70	26.3	20
Fat	4.3	10	34.9	60
kcal/g	3.85		5.24	
	**g**	**kcal**	**g**	**kcal**
Casein 80-mesh	200	800	200	800
L-Cystine	3	12	3	12
Cornstarch	315	1260	0	0
Maltodextrin 10	35	140	125	500
Sucrose	350	1400	68.8	275.2
Cellulose BW200	50	0	50	0
Soybean oil	25	225	25	225
Lard	20	180	245	2205
Mineral mix S10026	10	0	10	0
Dicalcium phosphates	13	0	13	0
Calcium carbonate	5.5	0	5.5	0
Potassium citrate	16.5	0	16.5	0
Vitamin mix V10001	10	40	10	40
Choline bitartrate	2	0	2	0
FD and C dye	0.05	0	0.05	0

Supplementary Table 4. Primers sequences used in this study for qPCR.

Gene	Forward (5′ to 3′)	Reverse (5′ to 3′)
Pref-1	GGT CCC CTC TGT GAC AAG TG	CAA GTT CCA TTG TTG GCG CA
PPARγ	AGA CAT CAG CGC CTA CAT CG	GCT CCC GGG TAG TCA AAG TC
C/EBPα	TGG ACA AGA ACA GCA ACG AG	TCA CTG GTC AAC TCC AGC AC
FAS	GCT GCT GTT GGA AGT CAG C	AGT GTT CGT TCC TCG GCG TG
SCD-1	TGA CTA TCA TCA TGC CGG CC	CTT TGA CAG CCG GGT GTT TG
ACC	TGA CCG TGG GCA CAA AGT T	AGG AGG AAC CGC ATT TAT CGA
ATGL	CAA CGC CAC TCA CAT CTA CGG	GGA CAC CTC AAT AAT GTT GGC AC
HSL	TGC TCT TCT TCG AGG GTG AT	GAT GGC AGG TGT GAA CTG G
CPT-1α	GTG AAA AGC ACC AGC ACC TG	CAA ACA GTT CCA CCT GCT GC
ACOX1	ACT ACC TGG ACA GCC AAT GC	ATC AAG AAC CTG GCC GTC TG
ATPF-1	TG GCG ACA GGC TGG ACT CAG	GC TGC CCG AAG TCT TCT CAG CG
